# How can age-based vaccine allocation strategies be optimized? A multi-objective optimization framework

**DOI:** 10.3389/fpubh.2022.934891

**Published:** 2022-09-08

**Authors:** Hao Wu, Kaibo Wang, Lei Xu

**Affiliations:** ^1^Department of Industrial Engineering, Tsinghua University, Beijing, China; ^2^Vanke School of Public Health, Tsinghua University, Beijing, China

**Keywords:** infectious disease, SEIR model, multi-objective (MO) optimization, vaccine allocation, improved Strength Pareto Evolutionary Algorithm (SPEA2)

## Abstract

Human life is deeply influenced by infectious diseases. A vaccine, when available, is one of the most effective ways of controlling the spread of an epidemic. However, vaccine shortage and uncertain vaccine effectiveness in the early stage of vaccine production make vaccine allocation a critical issue. To tackle this issue, we propose a multi-objective framework to optimize the vaccine allocation strategy among different age groups during an epidemic under vaccine shortage in this study. Minimizing total disease onsets and total severe cases are the two objectives of this vaccine allocation optimization problem, and the multistage feature of vaccine allocation are considered in the framework. An improved Strength Pareto Evolutionary Algorithm (SPEA2) is used to solve the optimization problem. To evaluate the two objectives under different strategies, a deterministic age-stratified extended SEIR model is developed. In the proposed framework, different combinations of vaccine effectiveness and vaccine production capacity are investigated, and it is identified that for COVID-19 the optimal strategy is highly related to vaccine-related parameters. When the vaccine effectiveness is low, allocating most of vaccines to 0–19 age group or 65+ age group is a better choice under a low production capacity, while allocating most of vaccines to 20–49 age group or 50–64 age group is a better choice under a relatively high production capacity. When the vaccine effectiveness is high, a better strategy is to allocate vaccines to 65+ age group under a low production capacity, while to allocate vaccines to 20–49 age group under a relatively high production capacity.

## Introduction

Human development has been shaped by infectious diseases, and some infectious disease outbreaks have even led to the demise of a country, such as the biblical pharaonic plagues that hit Ancient Egypt in the middle of the Bronze Age at ~1715 BC, the plague in Athens from 430 to 425 BC that ended the golden age of Athens, and the Black Death bubonic plague in Europe in 1348, which killed millions of people (1). In recent decades, we have seen that SARS in 2003, H1N1 influenza in 2009, MERS in 2013, Ebola in 2014 and the COVID-19 since 2019 have led to millions of deaths, leading to substantial health and economic losses (2). Thus, the study of preventing an epidemic or eradicating an existing epidemic has been a hot topic in the area of public health.

Vaccination is one of the most effective medical methods to control or even eradicate the spread of a sudden epidemic. Over the course of more than two centuries of development, vaccines have helped eliminate epidemics such as smallpox and malaria, and markedly reduced the number of deaths from measles, tetanus and whooping cough each year. However, in the early stage of a vaccine being available, it is impossible to produce sufficient vaccine doses to vaccinate an entire population due to limited vaccine types and production capacity (2). Also, populations with different characteristics, such as age, sex, ethnic group, and occupation, may have different risks of being infectious or dying from a given disease (3, 4); for example, the elderly may have a high probability of becoming infected because most have weakened immune systems. Therefore, allocating limited vaccines over different populations to help more people heal from or avoid the disease entirely is a critical issue in epidemic control.

Many researchers have investigated vaccine allocation since the beginning of the 21st century. Particularly after the COVID-19 outbreak, vaccine development was put on the agenda early in various countries, and major breakthroughs were made quickly (5, 6). Many institutions and research groups began to focus on the optimization of the allocation of the COVID-19 vaccine. These studies can be divided into two types: qualitative and quantitative.

Among the qualitative studies, researchers suggested vaccination priorities based on ethical principles, economic principles, health principles, and so on. In September 2020, the WHO (7) proposed a framework for the allocation and prioritization of COVID-19 vaccination, whose global goal was to let vaccines equitably protect and promote human wellbeing among all people of the world. This framework primarily considered 6 principles: human will-being, equal respect, global equity, national equity, reciprocity and legitimacy. Persad et al. (8) also proposed suggestions, but the ethical values that they emphasized benefitted people and limited harm by prioritizing disadvantaged populations and equally concerning different populations of different genders. Roope et al. (9) focused on socioeconomic development and human health, and raised three different criteria: individual health benefits, social health benefits and economic benefits. A report by the US National Academics of Sciences, Engineering and Medicine (2) summarized the experience of some qualitative research on COVID-19 vaccine distribution and divided the principles to be followed into two categories: ethical principles, and procedural principles. Additionally, the report proposed a four-stage vaccine allocation strategy according to these principles.

Researchers who performed quantitative studies primarily used mathematical models to evaluate the outcomes (e.g., infection and death prevention) of different vaccine allocation strategies and then provided suggestions. Some researchers aimed to optimize vaccine allocation over an entire strategy space (2, 10–14), and others aimed to evaluate and compare pre-given strategies and choose the best one (15–17). Many researchers considered the optimal vaccine allocation over different age groups and thus used an age-stratified SEIR model as an epidemic simulation mathematical model; some also provided deeper considerations. For example, Matrajt et al. (14) and Mylius et al. (17) accounted for the risk of being infected and participated in the population according to age and risk in their models. Both also considered how the allocation time point during an epidemic affected the optimal allocation strategy. Preciado et al. (12) constructed a model at the individual level, used an arbitrary network aggregating an SIS epidemic model to simulate disease transmission in the population, and proposed a convex framework for optimal vaccine allocation over the network. In addition to age, optimal allocation over geographic regions was also studied. Yarmand et al. (13) considered a decision process in which vaccination is performed in two phases and formulated the vaccine allocation problem as a two-stage stochastic linear programming. The optimization objectives or comparison criteria that these studies considered were total infectious, total deaths, years of life lost, economic cost, level of intensive care unit need and so on (11). Even in a recent study of COVID-19 vaccine allocation, a similar research framework was used, for example, the studies of Matrajt et al. (14) and Bubar et al. (16), but the latter assumed that the vaccine may have different effectiveness in the different age groups and thus considered serological tests in their model.

To the best of our knowledge, although many studies have been conducted to solve the vaccine allocation problem when a vaccine shortage exists, and many evaluation criteria have been considered, few studies have considered the trade-off between different criteria, that is, considered multiple criteria simultaneously. In addition, most have considered prevention vaccine allocation or allocation once, but few have considered multistage vaccine allocation during an epidemic. Vaccine allocation is a long, multistage process, and strategies can vary at different stages when vaccine quantities are insufficient; thus, considering multiple stages is critical. The main focus of this research will be developing a framework for determining optimal multistage vaccine allocation strategies with multiple objectives.

In this study, we modeled the vaccine allocation problem as a two-objective optimization problem by considering the total number of disease onsets and the total number of severe cases simultaneously, and solved it with the SPEA2 algorithm. To evaluate the optimization goals, we developed an age-stratified deterministic extended SEIR model to simulate the spread of the disease. We extended the conventional SEIR model to a more complex type by considering more compartments. Using this model, the spread of an infectious disease can be simulated by adjusting certain model parameters. We also considered age-specific contact information and vaccine effects in the extended SEIR model. Additionally, we used a case study to describe the effectiveness of the proposed framework, optimizing the COVID-19 vaccine allocation strategy. Different production capacities, different vaccine effectiveness levels and different vaccine efficiency against death and severity were considered.

## Materials and methods

### Model formulation

We considered that there are *T* stages of vaccine allocation and divided the population into *M* vaccination groups, where *M* is not greater than the number of age groups (i.e., *N*). We used **X** = (*x*_11_, *x*_12_, …, *x*_1*M*_, …, *x*_*T*1_, …, *x*_*TM*_) to represent a vaccine allocation strategy, whose element *x*_*ti*_ is a representation of the proportion of the number of vaccines allocated to vaccination group *i* at the *t*-th vaccine allocation stage to the total number of available vaccines at the allocation stage. Specifically speaking, if the total available vaccine number is *M*_*t*_, then the number of vaccines allocated to vaccination group *i* is *M*_*ti*_ = *M*_*t*_ × *x*_*ti*_. *M*_*t*_ is related to the vaccine production capacity, which is calculated by the product of the vaccine production capacity percent and the population size in our case study. The vaccine allocation strategy among different age groups in the same vaccination group is that the number of vaccines allocated to each age group is proportional to its demands. The two objective functions, *f*(**X**) represents the total number of disease onsets, and *h*(**X**) represents the total number of severe cases (deaths and cases that require intensive care) under vaccine allocation strategy **X**. These functions do not have exact expression, and can be evaluated by the age-stratified deterministic extended SEIR model developed in this study. *f*(**X**) is the summation of the daily simulated new symptomatic infected individuals; and *h*(**X**) is the summation of the sum of the daily simulated new infected individuals requiring ICUs and the daily simulated new deaths. And detailed information about the model and how the strategy **X** is contained in the vaccination process simulation are described in the section Extended SEIR model. Using these notations, we developed the following optimizing programming model. The constraints mean that the total number of vaccine doses allocated cannot exceed the total number of available vaccines doses at the allocation stage. We used SPEA2 to solve this two-objective optimization problem. The process of SPEA2 and how we use it in this study is described in the section Optimization method:


$$\begin{array}{r} \min_{x}f\left(\text{X}\right)\\ \min_{x}h\left(\text{X}\right)\\ s.t.\;\overset{M}{\underset{i=1}{\sum }}x_{ti}\leq 1,\forall t\\ x_{ti}\geq 0,\;\forall i,t \end{array}$$

### Extended SEIR model

#### Model structure

**Figure 1:**
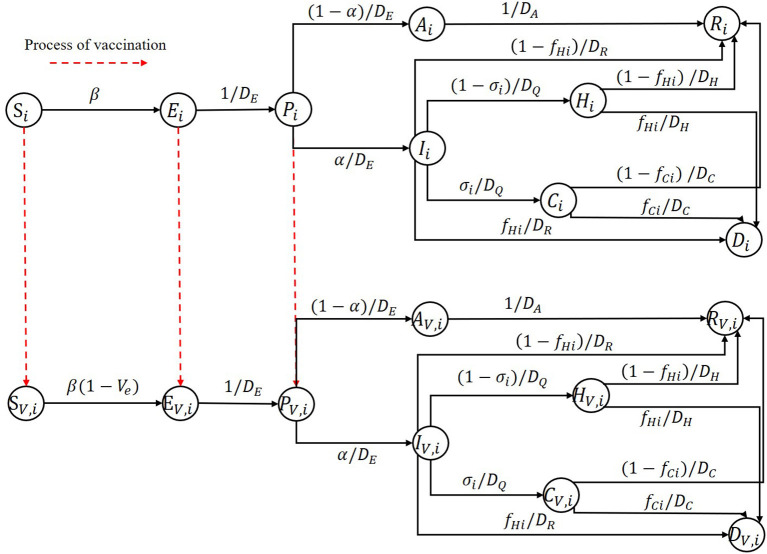
Illustration diagram of the extended age-stratified SEIR model.

We developed an age-stratified deterministic extended SEIR model that divides an entire population into N age groups by age. For each age group, as shown in Figure 1, the population is divided into 9 compartments: susceptible (S) population, exposed(E) population, pre-symptomatic (P) population, infectious population, recovered (R) population, and the people who died (D) from the epidemic. Pre-symptomatic individuals can become asymptomatic (A) individuals or symptomatic (I) individuals based on the severity of their symptoms. Those who are symptomatic may recover or die, require hospitalization (H), or require intensive care (C). A capital letter with the subscript V represents the corresponding population who has been vaccinated. The red dotted arrow represents the process of vaccination.

In this study, due to the short duration of the simulation, we did not consider any births or deaths caused by other reasons and assumed that the vaccines' effect last longer than the time horizon we study. Additionally, we assumed that different age groups had different infection fatality ratios, proportions of hospitalizations requiring ICU admission and susceptibilities. Other characteristics are held constant. Interventions other than vaccines, such as wearing masks and maintaining social distance, were also ignored in this study. We also assumed that one could not be confirmed as an infectious individual except based on for the onset of symptoms. Also, we assumed that at the early stage of vaccination, some testing methods, such as nucleic acid testing, were already mature and freely accessible to all people very easily. This is the real case in China for COVID-19; now almost all residents perform such tests regularly, which gives no chance for asymptomatic to get vaccine. Therefore, considering the scenario in China, we assumed that some tests were performed before one being vaccinated in our model, which can identify the asymptomatic infected individuals. Thus, they have no opportunity to get vaccine. Thus, the populations represented by S, E, and P have the opportunity to get vaccine, and we assumed that one could obtain immunity if and only if he or she gets two vaccine doses.

We described the social contact pattern between the age groups using a social contact matrix M. Its *i*-th row and *j*-th column element *m*_*ij*_ is the expectation value of the number of individuals in age group *j* that an individual in age group *i* contacts in a single day. All relevant parameters and their descriptions are shown in Table 1.

**Table 1:** Parameter descriptions of the extended age-stratified SEIR model.

**Parameters**	**Parameter description**	**Parameter value**	**Source**
M	Social contact matrix	See Figure 2	Zhang et al. (18)
*N*_*i*_	Size of the age group*i*	See Table 2	Calculation
*S*_*i*_	Size of the population *S* of the age group *i*, *E*_*i*_, *P*_*i*_, *A*_*i*_, *I*_*i*_, *C*_*i*_, *H*_*i*_, *R*_*i*_ and *D*_*i*_ mean the same.	–	Simulation
*r*_*P*_	Relative infectiousness of pre-symptomatic infectious	0.55	Hao et al. (19)
*r*_*A*_	Relative infectiousness of asymptomatic infectious	0.55	Hao et al. (19)
*D*_*E*_	Mean duration of latent period	2.9	Hao et al. (19)
*D*_*P*_	Mean duration of pre-symptomatic period	2.3	Hao et al. (19)
*D*_*A*_	Mean duration of asymptomatic period	2.9	Hao et al. (19)
*D*_*Q*_	Mean duration from illness onset to hospitalization	1.5	Assumption
*D*_*H*_	Mean duration of non-ICU hospitalization	25	Assumption
*D*_*C*_	Mean duration of ICU hospitalization	45	Assumption
α	Proportion of infectious that are symptomatic	0.15	Hao et al. (19)
*f*_*Ci*_	Infection fatality ratio of individuals requiring ICUs for age group*i*	See Table 2	Ferguson et al. (20)
*f*_*Hi*_	Infection fatality ratio of individuals not requiring ICUs for age group*i*	See Table 2	Ferguson et al. (20)
σ_*i*_	Proportion of hospitalization requiring ICU for age group*i*	See Table 2	Ferguson et al. (20)
*m*_*i*_	Relative susceptibility for those in age group*i*	See Table 2	Ferguson et al. (20)
β	Transmission coefficient	0.0528	Calculation
*D*_*R*_	Mean duration from illness onset to recovery or death without hospitalization.	25	Assumption
*R*_0_	Basic reproduction number	3	Wu et al. (21)
*V*_*e*_	Effectiveness of a vaccine	–	Setting
*V*_*ep*_	Vaccine efficiency against death and severity	–	Setting

Based on these assumptions and descriptions, the model equations without considering vaccination are:


$$\begin{array}{l} \frac{dS_{i}}{dt}=-\beta m_{i}S_{i}\underset{j}{\sum }\left(\frac{m_{ij}}{N_{j}}\left(r_{P}\left(P_{j}+P_{V,j}\right)+r_{A}\left(A_{j}+A_{V,j}\right)+\left(I_{j}+I_{V,j}\right)\right)\right) \end{array}$$

$$\begin{array}{l} \frac{dE_{i}}{dt}=\beta m_{i}S_{i}\underset{j}{\sum }\left(\frac{m_{ij}}{N_{j}}\left(r_{P}\left(P_{j}+P_{V,j}\right)+r_{A}\left(A_{j}+A_{V,j}\right)+\left(I_{j}+I_{V,j}\right)\right)\right)-\frac{E_{i}}{D_{E}} \end{array}$$

$$\begin{array}{l} \frac{dP_{i}}{dt}=\frac{E_{i}}{D_{E}}-\frac{P_{i}}{D_{P}} \end{array}$$

$$\begin{array}{l} \frac{dA_{i}}{dt}=\frac{\left(1-\alpha \right)P_{i}}{D_{P}}-\frac{A_{i}}{D_{A}} \end{array}$$

$$\begin{array}{l} \frac{dI_{i}}{dt}=\frac{\alpha P_{i}}{D_{P}}-\frac{I_{i}}{D_{R}}-\frac{I_{i}}{D_{Q}} \end{array}$$

$$\begin{array}{l} \frac{dC_{i}}{dt}=\frac{\sigma_{i}I_{i}}{D_{Q}}-\frac{C_{i}}{D_{C}} \end{array}$$

$$\begin{array}{l} \frac{dH_{i}}{dt}=\frac{\left(1-\sigma_{i}\right)I_{i}}{D_{Q}}-\frac{H_{i}}{D_{H}} \end{array}$$

$$\begin{array}{l} \frac{dR_{i}}{dt}=\frac{\left(1-f_{Hi}\right)I_{i}}{D_{R}}+\frac{\left(1-f_{Ci}\right)C_{i}}{D_{C}}+\frac{\left(1-f_{Hi}\right)H_{i}}{D_{H}}+\frac{A_{i}}{D_{A}} \end{array}$$

$$\begin{array}{l} \frac{dD_{i}}{dt}=\frac{f_{Hi}I_{i}}{D_{R}}+\frac{f_{Ci}C_{i}}{D_{C}}+\frac{f_{Hi}H_{i}}{D_{H}} \end{array}$$

$$\begin{array}{l} i=1,2,\ldots ,N \end{array}$$

We considered a leaky vaccine that can reduce susceptibility to infection and model it as a reduced probability of getting infectious when a susceptible individual contacts an infectious individual. This capability of reduced susceptibility due to vaccine is referred to as the effectiveness of a vaccine (*V*_*e*_) in this article. We assumed that *V*_*e*_ were age-independent. According to this assumption, the model equations for describing the population that obtain vaccinated have the same structure as model equations without considering vaccination except for the first and the second equations, and for the vaccinated population, the two different equations are:


$$\begin{array}{l} \frac{dS_{V,i}}{dt}=-\beta \left(1-V_{e}\right)m_{i}S_{V,i}\underset{j}{\sum }\left(\frac{m_{ij}}{N_{j}}\left(r_{P}\left(P_{j}+P_{V,j}\right)+r_{A}\left(A_{j}+A_{V,j}\right)+\left(I_{j}+I_{V,j}\right)\right)\right) \end{array}$$

$$\begin{array}{l} \frac{dE_{V,i}}{dt}=\beta \left(1-V_{e}\right)m_{i}S_{V,i}\underset{j}{\sum }\left(\frac{m_{ij}}{N_{j}}\left(r_{P}\left(P_{j}+P_{V,j}\right)+r_{A}\left(A_{j}+A_{V,j}\right)+\left(I_{j}+I_{V,j}\right)\right)\right)-\frac{E_{V,i}}{D_{E}} \end{array}$$

For the process of vaccination, we assumed that a susceptible individual acquired immunity once he or she is vaccinated, and then we can use the following equations to model the process:


$$\begin{array}{r} S_{V,i,t_{1}}=S_{V,i,{\left(t-1\right)}_{L}}+\min\left\{S_{i,{\left(t-1\right)}_{L}},\frac{S_{i{,\left(t-1\right)}_{L}}}{S_{{i,\left(t-1\right)}_{L}}+E_{i,{\left(t-1\right)}_{L}}+P_{{i,\left(t-1\right)}_{L}}}M_{ti}\right\}\\ E_{V,i,t_{1}}=E_{V,i,{\left(t-1\right)}_{L}}+\min\left\{E_{i,{\left(t-1\right)}_{L}},\frac{E_{i{,\left(t-1\right)}_{L}}}{S_{{i,\left(t-1\right)}_{L}}+E_{i,{\left(t-1\right)}_{L}}+P_{{i,\left(t-1\right)}_{L}}}M_{ti}\right\}\\ P_{V,i,t_{1}}=P_{V,i,{\left(t-1\right)}_{L}}+\min\left\{P_{i,{\left(t-1\right)}_{L}},\frac{P_{i{,\left(t-1\right)}_{L}}}{S_{{i,\left(t-1\right)}_{L}}+E_{i,{\left(t-1\right)}_{L}}+P_{{i,\left(t-1\right)}_{L}}}M_{ti}\right\}\\ S_{i,t_{1}}=S_{i,{\left(t-1\right)}_{L}}-\min\left\{S_{i,{\left(t-1\right)}_{L}},\frac{S_{i{,\left(t-1\right)}_{L}}}{S_{{i,\left(t-1\right)}_{L}}+E_{i,{\left(t-1\right)}_{L}}+P_{{i,\left(t-1\right)}_{L}}}M_{ti}\right\}\\ E_{i,t_{1}}=E_{i,{\left(t-1\right)}_{L}}-\min\left\{E_{i,{\left(t-1\right)}_{L}},\frac{E_{i{,\left(t-1\right)}_{L}}}{S_{{i,\left(t-1\right)}_{L}}+E_{i,{\left(t-1\right)}_{L}}+P_{{i,\left(t-1\right)}_{L}}}M_{ti}\right\}\\ P_{i,t_{1}}=P_{i,{\left(t-1\right)}_{L}}-\min\left\{P_{i,{\left(t-1\right)}_{L}},\frac{P_{i{,\left(t-1\right)}_{L}}}{S_{{i,\left(t-1\right)}_{L}}+E_{i,{\left(t-1\right)}_{L}}+P_{{i,\left(t-1\right)}_{L}}}M_{ti}\right\} \end{array}$$

where *M*_*ti*_ is the number of people who can obtain vaccinated using the vaccines allocated to age group *i* in the *t*-th time vaccine allocation, which is related to the vaccine production capacity, with two vaccine doses per person; *L* is the time lag between two adjacent vaccine allocation stages; *t*_1_ is the time point after the *t*-th time vaccine allocation; and (*t* − 1)_*L*_ is the time point before the *t*-th time vaccine allocation.

We take the first and the fourth equations as examples to explain the meaning of these eight equations. The first equation represents the immediate change of the size of vaccinated susceptible population *i* after the *t*-th time vaccine allocation. *S*_*V,i*,(*t*−1)_*L*__ is the size of vaccinated susceptible population *i* at the moment before *t*-th time vaccine allocation, and *S*_*V, i*,_*t*__1__ is the size of vaccinated susceptible population *i* immediately after *t*-th time vaccine allocation is completed. Under the assumption that the number of vaccines allocated to one compartment group is proportional to its size, $\min\left\{S_{i,{\left(t-1\right)}_{L}},\frac{S_{i{,\left(t-1\right)}_{L}}}{S_{{i,\left(t-1\right)}_{L}}+E_{i,{\left(t-1\right)}_{L}}+P_{{i,\left(t-1\right)}_{L}}+A_{{i,\left(t-1\right)}_{L}}}M_{ti}\right\}$ is the number of vaccines allocated to susceptible population *i* in the *t*-th time vaccine allocation. *min*{*a, b*} represents the smaller one between *a* and *b*, we used this notation to prevent the number of vaccines allocated to one compartment group to exceed its needs. And the fourth equation represents the immediate change of the size of unvaccinated susceptible population *i* after the *t*-th time vaccine allocation. Similarly, the other six equations represent the immediate change of the other compartment groups after the *t*-th time vaccine allocation.

#### Transmission coefficient calculation

Most disease parameters in the model, such as the mean duration of the latent period, can be determined from existing research about the disease, but the transmission coefficient β is not available. Due to policies and associated regularities made by the government, the social activity patterns of the population tend to change during an epidemic, which leads to a variational social contact matrix M; thus, we cannot model this variational pattern due to its complexity, and using real epidemic data to estimate β is nearly impossible.

This study aims to optimize the multi-time vaccine allocation strategy without considering other interventions; thus, we assumed that the M during an epidemic remained the same as it is in the normal situation, and the M in the normal situation was available because a few studies have given their estimation of M using some statistical methods based on some investigation data (18). We can thus use their results in this study. Many researchers have estimated the basic reproduction number *R*_0_ in the early stage of the epidemic due to its critical role in infectious disease studies, such as the *R*_0_ of COVID-19 (22). *R*_0_ has a tight relationship with nearly all the model parameters, including M and β. This reality inspired us to use all the other parameter values to calculate β directly. The next generation matrix (NGM) provided us with such a possibility. Using NGM to determine *R*_0_ for an ordinary differential equations (ODE) model was proposed by Diekmann et al. (23) and elaborated by Van den Driessche and Watmough (24). Thus, NGM helps us to construct a mathematical relation between *R*_0_ and model parameters. The following provides a brief introduction about how to construct an NGM and how to use it to calculate *R*_0_ and further displays the method of β calculation.

We assumed that the entire population was separated into *n* compartments and let **x** = (*x*_1_, *x*_2_, …, *x*_*n*_) be the number of individuals in each compartment. Without a loss of generality, we assumed that the first *m* < *n* compartments contained infectious individuals; then, the ODE of the first *m* compartments can be rewritten in the form of $\frac{dx_{i}}{dt}=F_{i}\left(\text{x}\right)-V_{i}\left(\text{x}\right)$, where $F_{i}\left(\text{x}\right)$ is the rate of appearance of new infections in compartment *i*, and $V_{i}\left(\text{x}\right)$ is the rate of other transitions between compartment *i* and other infected compartments. Using the concept of the Jacobian matrix, we defined $F={\left[\frac{dF_{i}\left({\text{x}}_{0}\right)}{dx_{j}}\right]}_{m\times m},\;V={\left[\frac{dV_{i}\left({\text{x}}_{0}\right)}{dx_{j}}\right]}_{m\times m}$, where **x**_0_ is a disease freedom equilibrium (DFE) point of the system. Then, *FV*^−1^ is the NGM of the model, and *R*_0_ is the spectral radius of the NGM.

In the proposed model, we assumed that compartments E, P, A, and I contained infectious individuals. According to the splitting principles mentioned above, the F functions, V functions, and DFE should be:

$$\begin{array}{l} F=\left(\frac{\beta m_{i}S_{i}\sum_{j}\frac{m_{ij}}{N_{j}}\left(r_{P}P_{j}+r_{A}A_{j}+I_{j}\right)}{\begin{array}{c} 0\\ 0\\ 0 \end{array}}\right),i=1,2,\ldots ,N \end{array}$$

$$\begin{array}{l} V=\left(\begin{array}{c} \frac{E_{i}}{D_{E}}\\ \frac{P_{i}}{D_{P}}-\frac{E_{i}}{D_{E}}\\ \frac{A_{i}}{D_{A}}-\frac{\left(1-\alpha \right)P_{i}}{D_{P}}\\ \frac{I_{i}}{D_{R}}+\frac{I_{i}}{D_{Q}}-\frac{\alpha P_{i}}{D_{P}} \end{array}\right),i=1,2,\ldots ,N \end{array}$$

$$\begin{array}{l} x_{0}=\left(S_{0,1\times N},E_{0,1\times N},P_{0,1\times N},A_{0,1\times N},I_{0,1\times N},H_{0,1\times N},\right)\\ \;{\left(C_{0,1\times N},\;R_{0,1\times N},\;D_{0,1\times N}\right)}^{T}\\ \;={\left(N_{1},N_{2},\ldots ,N_{N},0_{1\times 8N}\right)}^{T} \end{array}$$

Defining $M^{{}^{\prime }}={\left[m_{ij}^{{}^{\prime }}\right]}_{N\times N}={\left[\frac{m_{i}m_{ij}S_{i0}}{N_{j}}\right]}_{N\times N}$, we provide the formulation of *F* and *V* as follows:

$$\begin{array}{l} F=\left[\begin{array}{c} 0_{N\times N}\;\beta r_{P}M^{{}^{\prime }}\;\beta r_{A}M^{{}^{\prime }}\;{\beta M}^{{}^{\prime }}\\ 0_{N\times N}\;0_{N\times N}\;0_{N\times N}\;0_{N\times N}\\ 0_{N\times N}\;0_{N\times N}\;0_{N\times N}\;0_{N\times N}\\ 0_{N\times N}\;0_{N\times N}\;0_{N\times N}\;0_{N\times N} \end{array}\right]\\ =\beta \left[\begin{array}{c} 0_{N\times N}\;r_{P}M^{{}^{\prime }}\;r_{A}M^{{}^{\prime }}\;M^{{}^{\prime }}\\ 0_{N\times N}\;0_{N\times N}\;0_{N\times N}\;0_{N\times N}\\ 0_{N\times N}\;0_{N\times N}\;0_{N\times N}\;0_{N\times N}\\ 0_{N\times N}\;0_{N\times N}\;0_{N\times N}\;0_{N\times N} \end{array}\right]=\beta F^{{}^{\prime }} \end{array}$$

$$\begin{array}{l} V=\left[\begin{array}{c} \frac{1}{D_{E}}I_{N\times N}\;0_{N\times N}\;0_{N\times N}\;0_{N\times N}\\ -\frac{1}{D_{E}}I_{N\times N}\;\frac{1}{D_{P}}I_{N\times N}\;0_{N\times N}\;0_{N\times N}\\ 0_{N\times N}\;-\frac{\left(1-\alpha \right)}{D_{P}}I_{N\times N}\;\frac{1}{D_{A}}I_{N\times N}\;0_{N\times N}\\ 0_{N\times N}\;-\frac{\alpha }{D_{P}}I_{N\times N}\;0_{N\times N}\;\left(\frac{1}{D_{R}}+\frac{1}{D_{Q}}\right)I_{N\times N} \end{array}\right] \end{array}$$

where **0**_*N* × *N*_ is a zero matrix with dimensions of *N* × *N*, and *I*_*N* × *N*_ is an identity matrix with dimensions of *N* × *N*. We used ρ to represent the spectral radius of a matrix. Then, the following mathematical relation yields us a method to calculate β by other model parameters and *R*_0_:

$$\begin{array}{l} R_{0}=\rho \left(FV^{-1}\right)=\beta \rho \left(F^{{}^{\prime }}V^{-1}\right) \end{array}$$

#### Model extension

**Figure 2:**
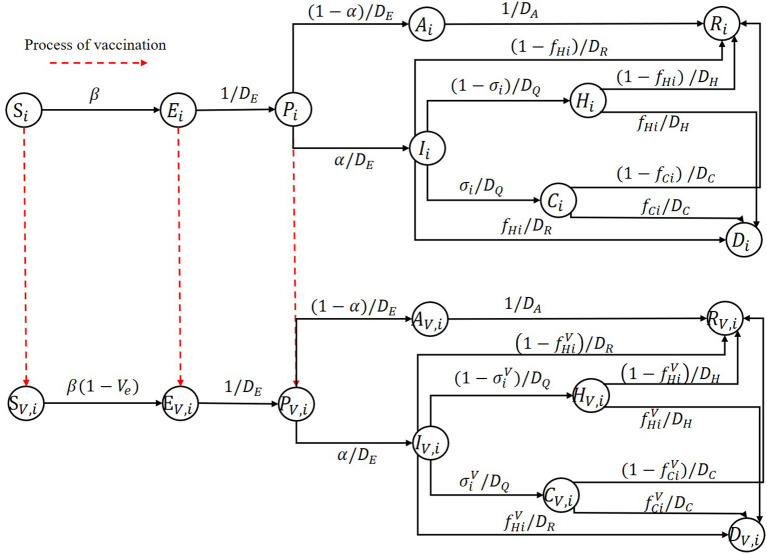
Illustration diagram of the further extended age-stratified SEIR model.

We discuss an important problem in this subsection, which may significantly influence the optimal vaccine allocation results. Some vaccines not just protect one individual from being infected, but reduce the infected fatality rate and the proportion of the hospitalization individuals requiring ICU. To tackle this important issue, we further extended our SEIR model, the model structure is shown in Figure 2. To represent the vaccine efficiency against death and severity, we added a parameter *V*_*ep*_ in the SEIR model. Then the infected fatality rate and the proportion of hospitalization individuals requiring ICU of vaccinated group are $f_{C\left(H\right)i}^{V}={\left(1-V_{ep}\right)f}_{C\left(H\right)i}$ and $\sigma_{i}^{V}=\left(1-V_{ep}\right)\sigma_{i}$, respectively. The model equations for describing the population that obtain vaccinated in this scenario have the same structure as model equations considering vaccination in section Model structure except for replacing *f*_*C*(*H*)*i*_ and σ_*i*_ with $f_{C\left(H\right)i}^{V}$ and $\sigma_{i}^{V}$.

### Optimization method

In this study, we considered two optimization objectives simultaneously: the total number of onsets and the total number of severe results (cases that require intensive care and deaths). We then optimized the vaccine allocation strategy with different vaccine effectivenesses, production capacities and efficiency against death and severity.

We must solve a two-objective optimization problem. The methods used to solve a multi-objective problem can be divided into two types (25, 26). The first type is to transform the original optimization problem into a single objective optimization problem and then to solve the new problem by some single-objective optimization method; however, these methods may have some strong restrictions on the property of objective space. The second type is to solve the original problem directly and to find a set of optimal solutions named Pareto optimal solutions; however, these methods cannot typically find all true Pareto optimal solutions and only obtain a set of nondomination solutions as an approximation. Almost all of these methods are evolutionary methods and are used widely in the area of multi-objective optimization; however, these methods are time-consuming. In this study, we used the Improved the Strength Pareto Evolutionary Algorithm (SPEA2), which was proposed by Zitzler et al. (27). Based on the Strength Pareto Evolutionary Algorithm (SPEA), Algorithm 1 shows the framework of SPEA2, and more details of SPEA2 are available in the reference paper.

**Algorithm 1:** SPEA2 framework.

**Input:** *N* (population size); $\bar{N}$ (archive size); *T* (maximum number of generations)
**Output:** *D* (nondomination set)
Step 1: ***Initialization***: Generate an initial population *P*_0_ and create the empty archive (external set) $\bar{P_{0}}=\phi$. Set *t* = 0.
Step 2: ***Fitness assignment***: Calculate fitness values of individuals in *P*_*t*_ and $\bar{P_{t}}$.
Step 3: ***Environment selection***: Copy all nondominated individuals in *P*_*t*_ and $\bar{P_{t}}$ to $\bar{P_{t+1}}$, if size of $\bar{P_{t+1}}$ exceeds $\bar{N}$ then reduce $\bar{P_{t+1}}$ by an exact truncation operator, otherwise if size of $\bar{P_{t+1}}$ is less than $\bar{N}$ then fill $\bar{P_{t+1}}$ with dominated individuals in *P*_*t*_ and $\bar{P_{t}}$.
Step 4: ***Termination***: if *t* ≥ *T* or another stopping criterion is satisfied then set *D* to the set of decision vectors represented by the nondominated individuals in $\bar{P_{t+1}}$. Stop.
Step 5: ***Mating selection***: Perform binary tournament selection with replacement on $\bar{P_{t+1}}$ to fill the mating pool.
Step 6: ***Variation***: Apply recombination and mutation operators to the mating pool and set $\bar{P_{t+1}}$ to the resulting population. Increment generation counter (*t* = *t* + 1) and go to Step 2.

An individual of SPEA2 has the same structure as the allocation strategy **X** (decision variables) that we defined in the section Model formulation, and each element of the vector **X** is a gene of the related individual; thus, the individual is real-coded in this problem. Thus, we used extended intermediate crossover (28) and Mühlenbein's mutation (28) in Step 6.

Note that SPEA2 cannot guarantee the feasibility of a solution, and the programming problem that we formulated in the Section Model formulation has two types of constraints. Infeasible solutions may be generated in Step 6 that must be ‘repaired’. First, if there are some *x*_*ij*_s in an individual being < 0, we must generate a random number from a uniform distribution between 0 and 1 to replace it. Then, if a solution **X** with $\overset{N}{\underset{i=1}{\sum }}x_{ti}>1$ for some *t*, we must map it to **X'** such that $\overset{N}{\underset{i}{\sum }}x_{ti}^{{}^{\prime }}>1$ for those *t* by replacing *x*_*ti*_ with $x_{ti}^{{}^{\prime }}=x_{ti}/\overset{N}{\underset{i=\;1}{\sum }}x_{ti}$.

There may also be another type of potentially infeasible situation. Even if a solution meets the constraints of the programming model, it may mislead the analysis and may yield confusing or incorrect suggestions. This type of situation is where vaccines that are allocated to some age groups at some vaccine allocation times exceed their total demands at that time; thus, a portion of vaccines are wasted. By replacing the proportion with the true demand proportion, we can ‘repair’ such an infeasible situation.

## Results

### Simulation settings

We applied the framework proposed in this study to COVID-19 vaccine allocation in a population whose age structure is similar to Wuhan City, China. The COVID-19 epidemic has led to tens of millions of deaths and heavily impeded economic development (29). A number of modelers have made efforts to develop a variety of models to study the spread of the epidemic (19, 30–39). These studies have provided adequate information on the characteristics of the infectious disease, such as the basic reproduction number and duration of the latent period (22). Most parameter values of the simulation model we used in this section were borrowed from previous studies (Table 1). It should be noted that the infected fatality rate of individuals requiring ICUs is greater than that of not requiring ICUs. However, we treated deaths and cases requiring ICUs both as severe cases, thus, the death rate for individuals requiring ICUs is not so important in this study. In addition, the overall death rate approximately equals the death rate of individuals requiring hospitalization due to the relatively low severity rate of COVID-19 (1). Hence, we assumed *f*_*Ci*_ = *f*_*Hi*_ = *f*_*i*_, and used the overall death rate to approximate the death rate of individuals requiring hospitalization in our case study, which is reasonable.

**Figure 3:**
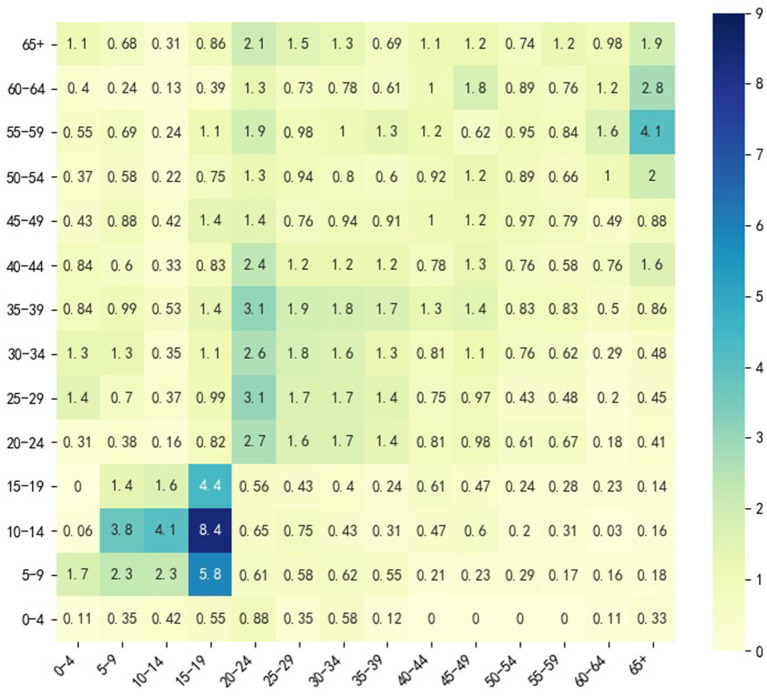
Heatmap of M in Wuhan City.

Since we wanted to simulate the spread of COVID-19 among different age groups without any other interventions except vaccine injection, a social contact matrix M was required, which can describe the contact pattern of the population mentioned above in the normal situation. We used the M obtained by Zhang et al. (18) because it described the social contact patterns of citizens in Wuhan from December 24, 2019, to December 30, 2019, when citizens of Wuhan City had no sense of the outbreak of the epidemic. M divides the entire population into 14 age groups (*N* = 14): 0–4, 5–9, 10–14, 15–19, 20–24, 25–29, 30–34, 35–39, 40–44, 45–49, 50–54, 55–59, 60–64, and 65+; Figure 3 shows a heatmap of M. For the 65+ age group, we performed a weighted average of the rates in the Table 2 according to the relative percentages of the population aged 65–69, 70–74, 75–79, and 80+.

**Table 2:** Values of *N*_*i*_, *f*_*i*_, σ_*i*_, and *m*_*i*_ of different age groups.

**Age group (*i*)**	***N*_*i*_**	***f*_*Ci*_ & *f*_*Hi*_**	**σ_*i*_**	***m*_*i*_**
0–4	601,242	0.002%	5.0%	0.34
5–9	496,533	0.002%	5.0%	0.34
10–14	508,968	0.006%	5.0%	0.34
15–19	969,706	0.006%	5.0%	1
20–24	1,126,637	0.03%	5.0%	1
25–29	811,051	0.03%	5.0%	1
30–34	790,500	0.08%	5.0%	1
35–39	980,104	0.08%	5.0%	1
40–44	1,133,132	0.15%	6.3%	1
45–49	1,030,689	0.15%	6.3%	1
50–54	746,320	0.6%	12.2%	1
55–59	744,283	0.6%	12.2%	1
60–64	559,280	2.2%	27.4%	1
65+	713,556	4.6%	41.0%	1.47

We obtained the population size of each age group in Hubei Province from the Sixth Nation Population Census in 2010 from the official website of the National Bureau of Statistics of China (40) and estimated the age structure of Wuhan citizens from the data. Aggregating with the population size of Wuhan in 2020, we calculated the size of each age group in the population that we considered to perform simulations while ignoring the age structure changes during the last decade. Note that the basic reproduction number we used to calculate β was estimated using the data from the early stage of the epidemic. We set *D*_*Q*_ = 21 days when calculating β, and we set *D*_*Q*_ = 1.5 days when performing simulation because the epidemic prevention and control is strict now, and the time lag between symptom onset and hospitalization is shorter than 2 days.

Figure 3 shows that in the normal situation, the highest social contacts occur between 5 and 19 age groups and their peers. The contact between 20 and 49 age group and their peers is also frequent, but the frequency is lower than that between 5 and 19 age groups and their peers. Individuals in 20–49 age group also frequently contact the 50–64 age group. The 50–64 age group contacts both the 20–49 age group and the 65+ age group frequently, which indicates that 20–49 and 50–64 age groups may have a higher transmission risk. This analysis inspired us to consider 4 vaccination groups (*M* = 4): 0–19 age group, 20–49 age group, 50–64 age group and 65+ age group. We considered 3 vaccine allocation stages (*T* = 3), and the time lag between any two adjacent allocation time points was 30 days (*L* = 30). To investigate how vaccine effectiveness and vaccine production capacity affect optimization, we considered 9 vaccine effectiveness (*V*_*e*_ from 10 to 90% in steps of 10%) and 5 vaccine production capacities (VPCs) (5, 10, 15, 20, 25%). Vaccine production capacity was defined as the proportion of the number of vaccine doses available at each allocation time point over the size of the entire population that we considered, and we assumed that the capacity was the same at different stages. We only set *V*_*ep*_ = 0, 0.3, 0.6, 0.9 when *V*_*e*_ = 0.3, 0.6, 0.9 in the model extension. In this section, we assumed that all individuals were susceptible; thus, the size of the population with pre-existing immunity was zero, and in real applications, this setting could be changed when different real scenarios are met.

The situation we considered in this section was an imported epidemic, where several infected individuals are introduced into an epidemic-free area and lead to an epidemic outbreak. Thus, the initial simulation state we set was one infected individual in each age group, and the others were all susceptible individuals. The time length we considered to calculate the total onsets and severe cases was 200 days. Additionally, the baseline strategy we used to compare with optimal strategies was that the number of vaccines allocated to each age group is proportional to its demands. Under the above settings, the optimal allocation strategies were obtained using SPEA2 algorithm descripted in section Optimization method.

### Optimization results analysis

To display and analysis the final optimization results, we introduce some basic concepts in the field of multi-objective optimization (41):

- Pareto domination: A solution **X** dominates another solution **Y** if all the objectives of **X** are not worse than **Y**'s, and **X** is strictly better than **Y** in at least one objective.
- Pareto optimality: A solution **X** is a Pareto optimal solution if there is no solution in the feasible region dominating **X**.
- Global Pareto optimal set: A global Pareto optimal set consists of all the Pareto optimal solutions in the feasible region.
- Pareto optimal front: The projections of all the elements of the global Pareto optimal set in the objective space constitute the Pareto optimal front.

Solving a multi-objective problem requires identifying its Pareto optimal front; however, finding the true Pareto optimal front is generally impossible. Thus, the primary aim of solving a multi-objective optimization problem is to find a well-distributed, approximate Pareto optimal front. A Pareto figure is a tool to display the optimization results in the field of multi-objective optimization. Usually, the two axises represent different objective function values; points on the figure represents the projections of feasible (or Pareto optimal) solutions in the objective space.

**Figure 4:**
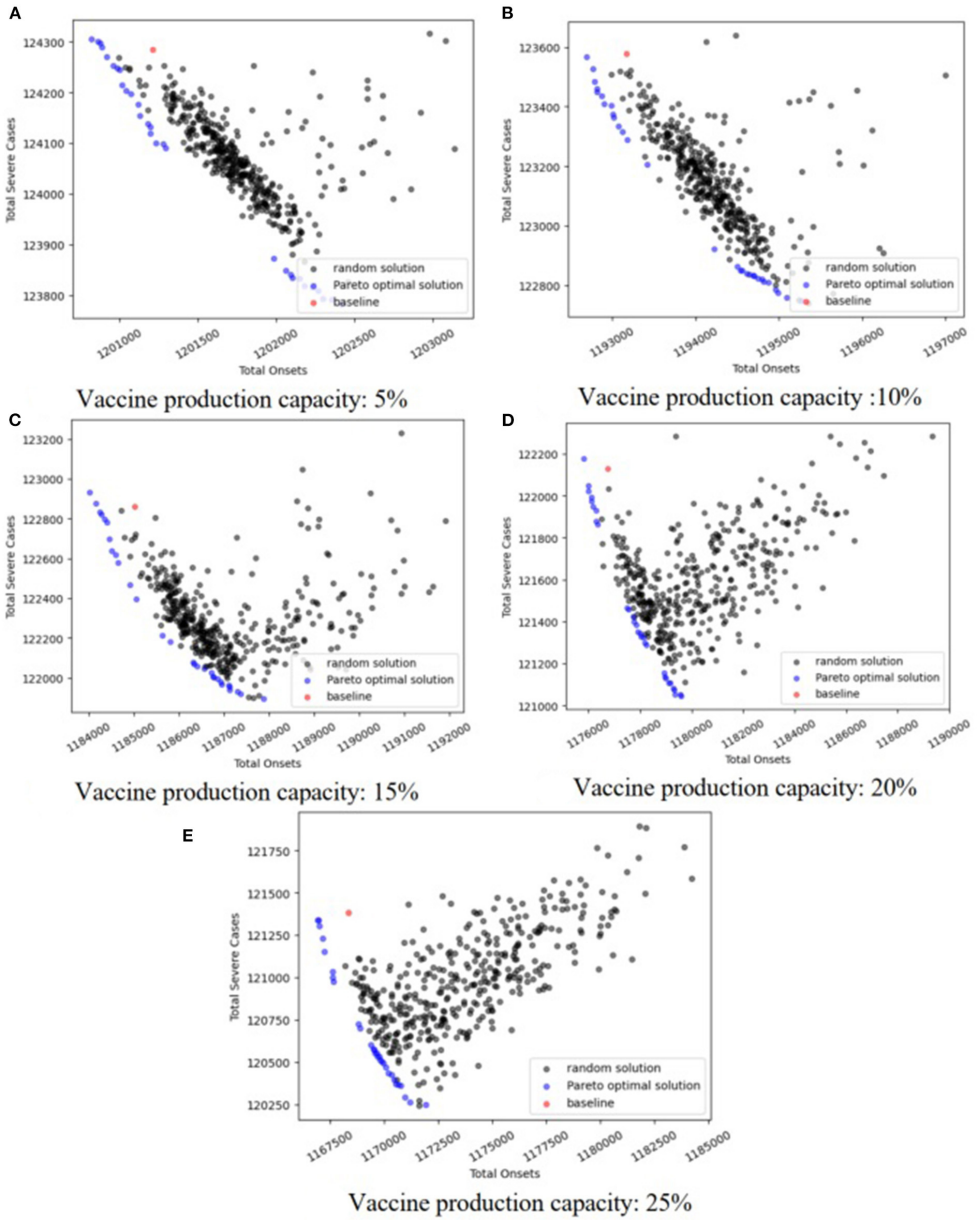
Pareto optimal fronts when *V*_*e*_ = 10%.

**Figure 5:**
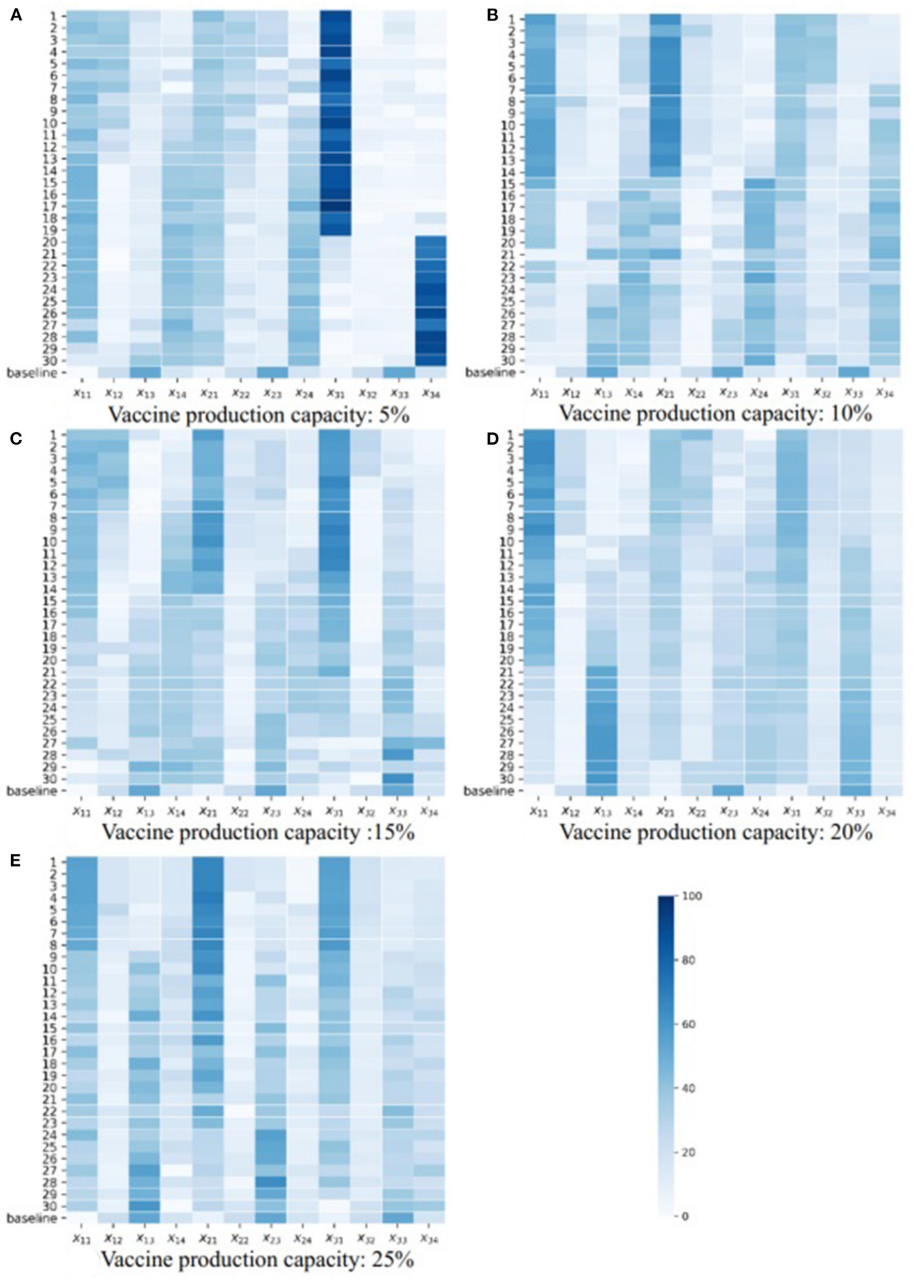
Heatmaps of Pareto optimal solutions when *V*_*e*_ = 10%.

We drew Pareto figures under the different combinations of vaccine effectiveness and vaccine production capacity (Figure 4; Supplementary Figures S1–S8). The subfigures A–E in Figures 4, 5 are the Pareto figures under different vaccine production capacities, and the relevant vaccine production capacity is displayed below each subfigure. In each subfigure, the abscissa axis is the total severe cases (*h*(**X**)), and the ordinate axis is the total onsets (*f*(**X**)). Different subfigures in each figure show the results under different vaccine production capacities. The black points in each figure are the simulation results of 800 random strategies; the blue points are the approximate Pareto optimal front obtained by SPEA2, the simulation outputs of the Pareto optimal solutions; and the red point is the simulation output of the baseline strategy. We could easily notice that the black points and the red points always lie on the upper right side of the approximate Pareto optimal front, which means that the optimization process is important. Also, in the scope of this study, along with the increase in vaccine effectiveness and vaccine production capacity, we discovered that the contradiction between the two objectives becomes weaker, and the positive correlation property becomes stronger. Consequently, the Pareto optimal front is broad in a low vaccine effectiveness (not > 30%, Figure 4; Supplementary Figures S1, S2) situation and narrow with relatively high vaccine effectiveness (not < 70%, Supplementary Figures S6–S8), while with a medium vaccine effectiveness (say, between 30 and 70%, Supplementary Figures S3–S5), the Pareto optimal front is broad under a low vaccine production capacity and narrow under a relatively high vaccine production capacity.

In an effort to explore the detailed structure of the Pareto solutions and how the vaccine parameters affect the Pareto solutions, we drew a heatmap of the Pareto solutions under each combination of vaccine parameters (Figure 5; Supplementary Figures S9–S16). The subfigures A–E in Figures 4, 5 are the heatmap of the Pareto solutions under different vaccine production capacities, and the relevant vaccine production capacity is displayed below each subfigure. Each row of each heatmap is a corresponding Pareto solution, and the number of its left side is the solution id. The solution numbered ‘baseline’ is the baseline solution. In each heatmap, the direction of solution id increasing is the direction of the total onsets increasing but total severe cases decreasing. The color of each square is the related value of *x*_*ti*_.

The structure of the baseline solution indicates that the baseline strategy always allocates most vaccines to the 20–49 age group, allocates the second most vaccines to the 0–19 age group, and allocates the fewest number of vaccines to the 65+ age group, regardless of vaccine parameters and allocation stage when the vaccine is insufficient. This baseline strategy strongly positively correlates with the age group size, which is consistent with our perceptual intuition.

Based on the heatmaps of the Pareto optimal solutions, we summarized the rules described in the optimal allocation strategy under different vaccine effectiveness and vaccine production capacity. These rules were only discussed within the value range of each parameter considered in this case study.

If we look at the 0–49 age group as a whole, and the 50+ age group as a whole, and consider the three vaccine allocation phases together, the optimal strategies that tend to minimize the total number of onsets suggest allocating most of the vaccines to the 0–49 age group, while optimal strategies that tend to minimize the total number of severe results (i.e., deaths or ICU cases) suggest allocating most of the vaccines to the 50+ group. This tendency diminishes as vaccine effectiveness increases and vaccine production capacity increases. When the vaccine effectiveness and the vaccine production capacity reach a certain level, the contradiction between the two goals is weakened, the positive correlation is enhanced, the Pareto front becomes narrow, we can only obtain a small number of Pareto optimal solutions, and this trend tends to disappear.

Note that people aged 65+ have a higher risk of being infected and dying from the infectious, and we referred the strategy that directly allocates most of the vaccines to this population as the direct protection of a high-risk population. Conversely, although young and middle-aged groups have a lower rate of infection and death, they frequently contact most age groups and thus have a higher transmission risk. Protecting these age groups can control epidemic spread, and we thus referred the strategies that directly protect these age groups as the indirect protection of high-risk populations.

Based on this analysis, we provided a more detailed discussion. When the vaccine effectiveness is low (10, 20, and 30%, Figure 5; Supplementary Figures S9, S10), and with increasing vaccine production capacity, the vaccines allocated to the 0–49 age group gradually shift from being more allocated to the 0–19 age group to being more allocated to the 20–49 age group. Also, the vaccines allocated to the 50+ age group gradually shift from being more allocated to the 65+ age group to the 50–64 age group (i.e., shifting from direct protection to indirect protection). The results in each allocation stage are consistent. When the vaccine effectiveness is relatively high (60, 70, 80, and 90%, Supplementary Figures S13–S16), and the vaccine production capacity is low (5%), the optimal strategy suggests allocating most of the vaccine to the 65+ age group to directly protect them. As vaccine production capacity increases, the optimal strategy suggests allocating most of the vaccines to the 20–49 and 50–64 age groups. The strategy with a higher vaccine effectiveness in this range tends to allocate more vaccines to the 20–49 age group and fewer to the 50–64 age group. This trend is enhanced when the allocation stage increases. Additionally, with medium vaccine effectiveness (40 and 50%, Supplementary Figures S11, S12), and when vaccine production capacity is low (5%), the optimal strategy allocates most vaccines to the 20–49 or 65+ age group according to the optimizing tendency, and the results at each allocation stage are consistent. As vaccine production capacity increases, the optimal strategy allocates a proportion of vaccines to the 65+ age group, which decreases as the allocation stage increases and then primarily allocates the vaccines to the 20–49 or 50–64 age group in line with the optimization tendency. When the vaccine effectiveness and vaccine production capacity are sufficiently high (90%, 25%, Supplementary Figure S16E), the optimal strategy primarily allocates vaccines to 20–49 and 65+ age groups, and with increasing allocation, the proportion allocated to 20–49 age group increase, while the proportion allocated to 65+ age group decrease.

### Simulation comparation of optimal strategy and baseline strategy

To evaluate the optimal strategy in more detail, we chose one solution for each combination of vaccine parameters to perform a simulation analysis and compare the simulation outcomes with the outcomes of the baseline strategy. Note that the difference in the magnitude of the two objectives is large. We scaled them to values between 0 and 1 using Equations (2) and (3), where **D** is the nondomination set obtained under a combination of vaccine parameters, and then choose the solution that is the closest to the original point after scaling (described as Equation 1) in the objective space. As shown in the Pareto figures, with some vaccine parameters, the number of Pareto solutions is not > 2 due to the positive correlation property between the two objectives. In this scenario, we chose one Pareto optimal solution randomly:

(1) $$\begin{array}{l} \underset{{\text{X}}_{i}\in \text{D}}{\min}\sqrt{{\left(h^{s}\left({\text{X}}_{i}\right)\right)}^{2}+{\left(f^{s}\left({\text{X}}_{i}\right)\right)}^{2}} \end{array}$$

(2) $$\begin{array}{l} h^{s}\left({\text{X}}_{i}\right)=\frac{h\left({\text{X}}_{i}\right)-\min\left\{h\left({\text{X}}_{j}\right)|{\text{X}}_{j}\in \text{D}\right\}}{\max\left\{h\left({\text{X}}_{j}\right)|{\text{X}}_{j}\in \text{D}\right\}-\min\left\{h\left({\text{X}}_{j}\right)|{\text{X}}_{j}\in \text{D}\right\}} \end{array}$$

(3) $$\begin{array}{l} f^{s}\left({\text{X}}_{i}\right)=\frac{f\left({\text{X}}_{i}\right)-\min\left\{f\left({\text{X}}_{j}\right)|{\text{X}}_{j}\in \text{D}\right\}}{\max\left\{f\left({\text{X}}_{j}\right)|{\text{X}}_{j}\in \text{D}\right\}-\min\left\{f\left({\text{X}}_{j}\right)|{\text{X}}_{j}\in \text{D}\right\}} \end{array}$$

**Figure 6:**
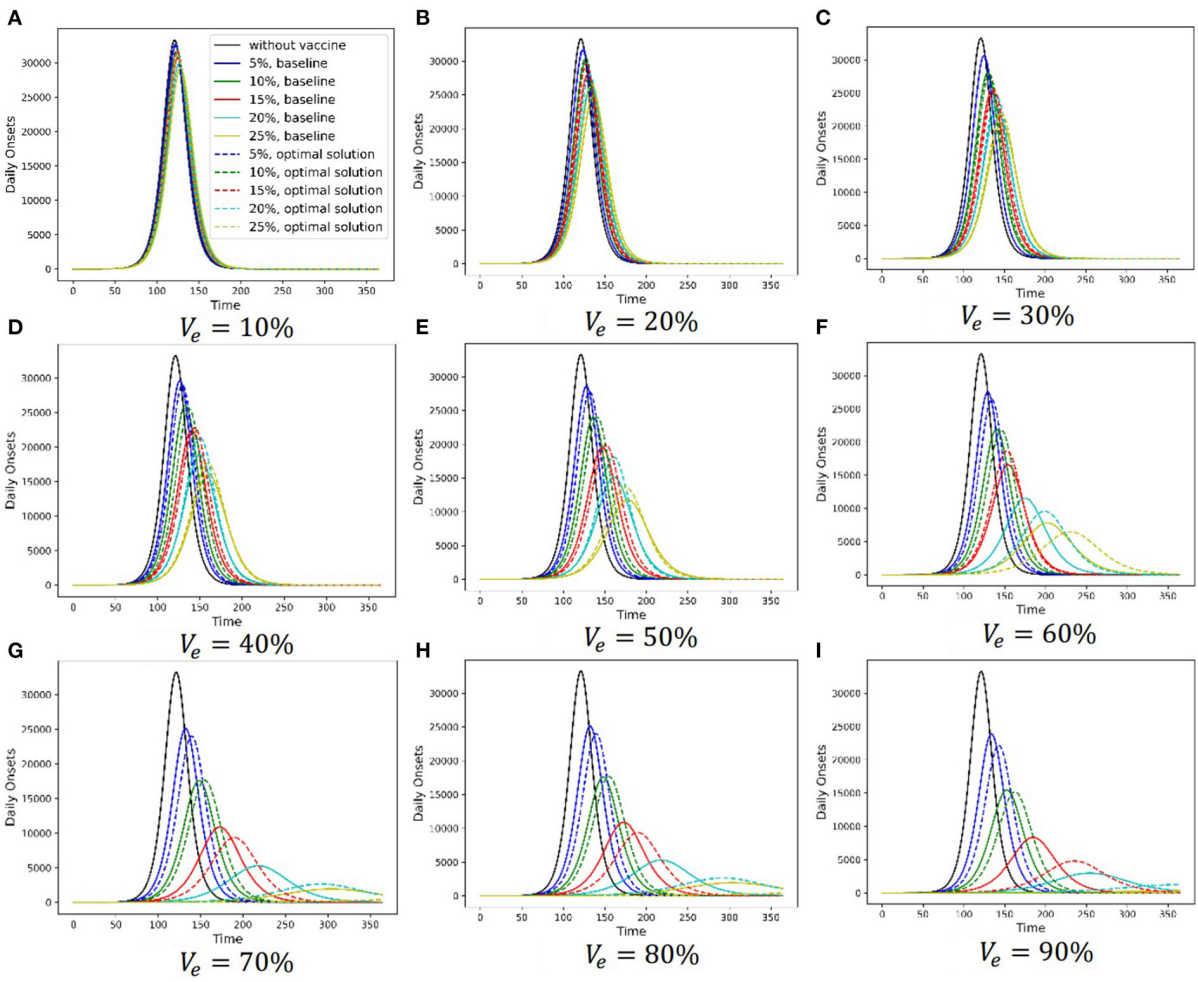
Simulation comparation between the chosen optimal strategy and baseline.

Figure 6 shows the simulation results of the baseline strategy and the chosen Pareto optimal strategy under different vaccine parameter combinations. Different subfigures are the results under different vaccine effectiveness, and relevant vaccine effectiveness is shown in Figures 6A–I. The abscissa axis is the simulation time, and the ordinate axis is the daily onset number. In each subfigure, the black solid line is the simulation result without a vaccine, the lines in other colors are the simulation results with a vaccine, and different colors stand for different vaccine production capacities. Solid lines show the baseline strategy simulation results, and dashed lines show the chosen Pareto optimal strategy simulation results. There are two primary findings from these simulations. First, vaccination can delay the arrival of the peak daily onset number and suppress it. Additionally, the ability of vaccination to delay and suppress the peak is enhanced as the two vaccine parameters increase. Second, the ability of the Pareto optimal strategy to delay and suppress the peak is more permanent than that of the baseline strategy.

**Figure 7:**
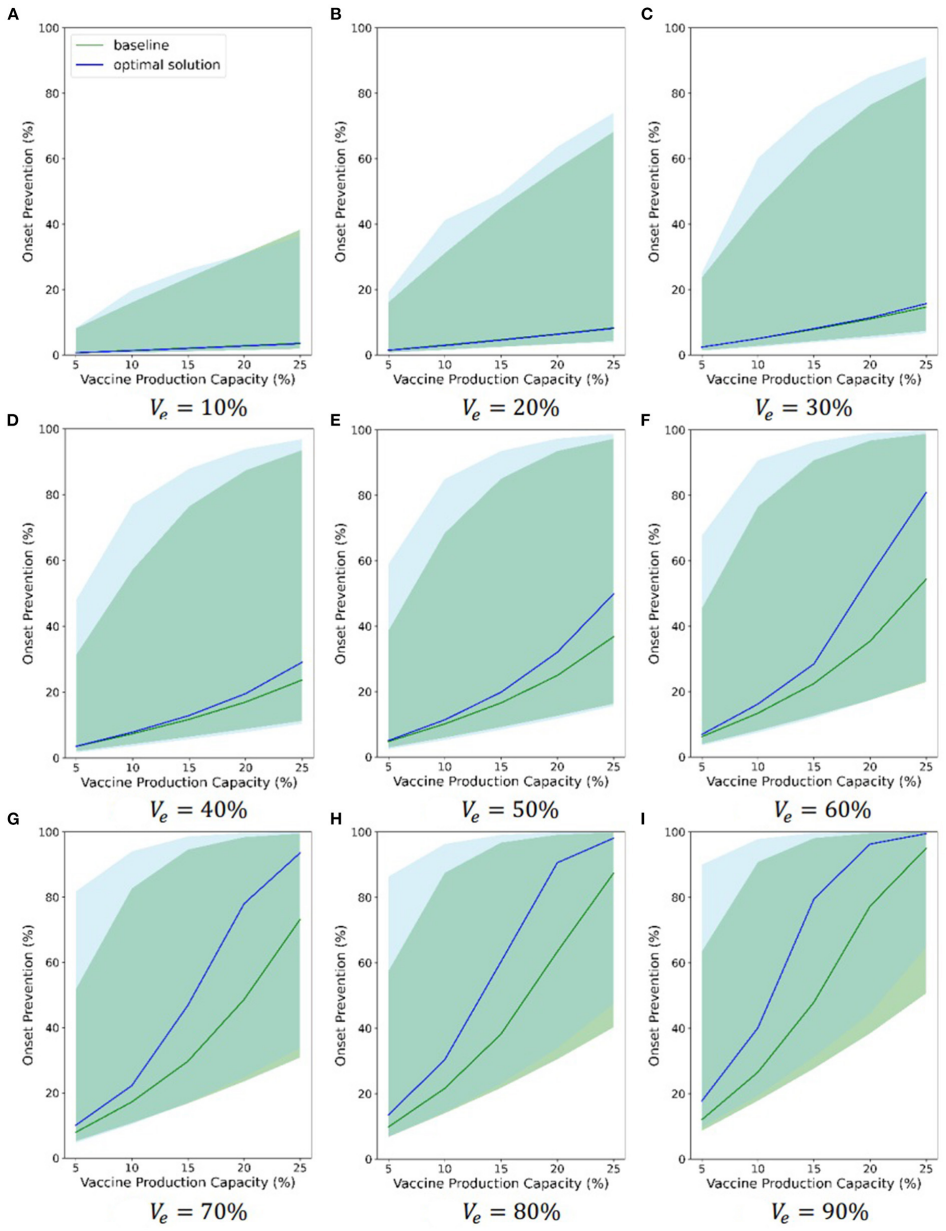
Onsets prevention comparation between chosen optimal strategy and baseline.

Also, considering the uncertainty of some model parameters, we randomized some model parameters (*R*_0_, α, *D*_*E*_, *D*_*P*_, *D*_*C*_, *D*_*H*_, *D*_*R*_, and *D*_*Q*_) and assigned a continuous probability distribution to each of these parameters. Then, we sampled 1,000 groups of parameters from these distributions. Finally, we ran 1,000 simulations using these parameters. Figure 7 and Supplementary Figures S17, S18 compare the chosen Pareto optimal strategy and baseline strategy with regard to onset prevention, ICU case prevention and death prevention separately under different combinations of vaccine parameters, respectively. Different subfigures show the results under different vaccine effectiveness, and relevant vaccine effectiveness is in Figures 6A–I. The abscissa axis is the vaccine production capacity, and the ordinate axis is the percentage of onset prevention, ICU case prevention or death prevention. The blue line shows the results of the chosen Pareto optimal strategy, and the green line shows the results of the baseline strategy. The shaded area of the corresponding color shows the range of 1,000 simulations without considering the least 2.5% and the most 2.5% simulations.

The figures indicate that both the baseline strategy and the chosen optimal strategy can prevent onsets, ICU cases and deaths, even if the least effective vaccine (10%) and the least production capacity (5%) are considered. The chosen Pareto optimal strategy is always better than the baseline strategy in all three evaluations. As the two vaccine parameters increase, the effectiveness of vaccines in preventing onsets, ICU cases and deaths becomes increasingly strong, and the advantages of the chosen Pareto optimal strategies become increasingly strong to some extent. When the vaccine is 80% effective and the vaccine production capacity exceeds 20%, the percentage of onset prevention, ICU case prevention and death prevention of the chosen strategy approaches or exceeds 90%. The range between the 2.5% limit and the 97.5% limit of 1,000 simulations is large, which indicates that the effectiveness of the Pareto optimal strategy is sensitive to the model parameters. Although the blue and green shaded areas overlap, the 2.5 and 97.5% limits of the chosen Pareto optimal strategy are nearly always greater than those of the baseline strategy separately, which highlights the advantages of the chosen optimal strategies.

### Optimization results analysis of extended model

We accounted for the vaccine that can reduce the infected fatality rate and the proportion that the hospitalization individuals requiring ICU. As is shown in section Model extension, we used a parameter *V*_*ep*_ to model this effect. We reoptimized the allocation policies under different combinations of *V*_*e*_ (30, 60, 90%), vaccine production capacity (VPC) (5, 10, 15, 20, 25%), and *V*_*ep*_ (0.3, 0.6, 0.9), analyzed the results and compared them with the results obtained when *V*_*ep*_ = 0 (i.e., did not consider the influence of vaccine on the infected fatality rate and the proportion that the hospitalization individuals requiring ICU).

Supplementary Figures S19–S21 show Pareto optimal fronts under different combinations of *V*_*e*_, VPC, and *V*_*ep*_. Each figure displays the results of a value of *V*_*e*_; each subfigure displays the results of a value of VPC; and in each subfigure, scatter points of different shapes represent results under different *V*_*ep*_. For the results under each combination of parameters, the yellow scatter points in each figure are the simulation results of 800 random strategies; the blue scatter points are the approximate Pareto optimal front obtained by SPEA2, the simulation outputs of the Pareto optimal solutions; and the red scatter point is the simulation output of the baseline strategy. We could find that the total onsets are the same but the total severe cases are different for a given vaccine allocation policy, vaccine with a higher *V*_*ep*_ has a lower number of the total severe cases. Consequently, for each combination of VPC and *V*_*ep*_, the Pareto optimal front with a higher *V*_*ep*_ is lower in the vertical direction than the Pareto front with a lower *V*_*ep*_, but there is little difference in the horizontal direction. As VPC and *V*_*e*_ increasing, this effect is weakened, and these Pareto fronts get so close to each other that we cannot distinguish them from each other in the picture for high enough VPC and *V*_*e*_ (Supplementary Figure S21E). These findings may be explained by the following two reasons. Firstly, we assumed that the recovered, dead, and hospitalized individuals had no potential to infect others, and *V*_*ep*_ can only affect the size of these three populations. In other words, the infected pressure of susceptible population is the same under different *V*_*ep*_s. Thus, *V*_*ep*_ has no effect on the total onsets, but has an effect on the total severe cases under the same vaccine allocation strategy. Secondly, the optimal policies under a high VPC and *V*_*e*_ suggest to allocate most vaccine doses to the high-transmission population no matter what value of *V*_*ep*_ is (the last row of Supplementary Figure S24), whose infected fatality rate and proportion of the hospitalization requiring ICU are low. Therefore, most of the severe cases are from high-risk population, and changing *V*_*ep*_ have a slight influence on it.

Supplementary Figures S22–S24 show heatmaps of Pareto optimal solutions of model extension. Each figure displays the results of a value of *V*_*e*_, each row in a figure shows the results of the same VPC and different *V*_*ep*_s, and each column in a figure shows the results of the same *V*_*ep*_ and different VPCs. We could find that the patterns of optimal allocation policies under different *V*_*ep*_s (each row of each figure) are similar, which indicates that *V*_*ep*_ does not have any significant influence on the optimal allocation policies in the scope of this study.

## Discussion

This study demonstrated the use of an extended deterministic age-stratified modeling approach together with a multi-objective optimization framework to optimizethe vaccine allocation strategies, and considered the trade-off between the two objectives, i.e., minimizing total onset cases and minimizing total severe cases. We mainly accounted for how different vaccine efficiencies and different vaccine production capacities influence on the optimal policy.

We performed a case study of COVID-19 vaccine allocation to validate the efficiency of the proposed method. One basic finding is that the optimal policies tend to minimize total severe cases prioritize the older population, while the optimal policies tend to minimize total onset cases prioritize the younger population. This finding is in agreement with some previous works that evaluate or optimize the COVID-19 vaccine allocation policies (14, 16, 42–45). This is mainly because the infected fatality rate of COVID-19 is highly positive correlation with age, that is, the older one typically has a higher fatality rate. However, Sunohara et al. (46) suggested that prioritized younger generation was better in terms of deaths under an assumption of a linear relationship between lockdown intensity and acceptable economic loss, and that under an assumption of non-linear relationship, the old first policies were best considering small basic reproduction number. We believe that the reason for the difference in results is that we did not consider factors such as lockdown and economic effect, but only considered the effect of vaccine effectiveness and productivity on the optimization results. To some extent, this shows that in order to obtain more reasonable optimization results, more factors need to be considered according to the actual situation. The results also indicate that the conflict between the two objectives is weakened as the increasement of the vaccine efficiency and the vaccine production capacity because of the increasement of vaccine coverage. What's more, vaccine efficiency and vaccine production capacity are able to influence the switch pattern of priority age group when the optimization tendency shifts from minimizing total onsets to minimizing total severe cases according to the results. For instance, with a low vaccine efficiency and a low vaccine production capacity, the priority age group switches from 0–19 to 65+ when the optimization tendency shifts from minimizing total onsets to minimizing total severe cases; but with a low vaccine efficiency and a high vaccine production capacity, the priority age group switches from 20–49 to 50–64 when optimization tendency shifts. In reality, the other factor, such as the vaccine type (leaky vaccine or all-or-nothing vaccine), can also affect the switch pattern (16). Although we did not consider any other factors except for vaccine efficiency and vaccine production capacity, we provided a detailed description for how these two factors influence priority switch pattern.

The policies we considered in the study are dynamic, that is, the proportion of vaccines allocated to each age group changes as the epidemic progresses. Some researchers also discussed this type of policies in their studies (42, 45). These studies argued that high priority groups—by percent of group vaccinated—switched from one to another as the epidemic develops. Due to the limitations of the optimization method, we only considered three allocation periods, so our results did not show similar phenomena. But the dynamic policies can definitely improve the efficiency of vaccine use due to its flexibility (42).

In summary, within the scope of this study, when vaccine effectiveness is low, the better policy is to allocate most vaccines to the 0–19 age group or 65+ age group under a low production capacity according to the optimization tendency and allocate most vaccines to the 20–49 age group or 50–64 age group according to the optimization tendency under a relatively high production capacity. When vaccine effectiveness is high, the better policy is to allocate vaccines to the 65+ age group with a low production capacity and allocate vaccines to the 20–49 age group with a relatively high production capacity. Matrajt et al. (14) found that under high vaccine coverage, the optimal allocation for all objective functions shifted toward the high-transmission groups. If we treat 20–49 and 50–64 age groups as high-transmission groups, we obtained the similar pattern. However, Matrajt et al. (14) considered one-time vaccine allocation, while we considered multi-period vaccine allocation. In addition, considering the vaccine that can reduce infected fatality rate and proportion of hospitalization requiring ICU do not significantly affect the optimal policy, but can reduce total severe cases.

In the parameter uncertainty study, the 95% confidence intervals are wide, which indicates that the optimal policies may be sensitive to these key parameters. This inspires us to optimize vaccine allocation policy using as precise parameters as possible. Moreover, the existence of other Non-medical interventions may influences the contact pattern, and further influences optimization results, such as the results of Sunohara et al. (46) which considered lockdown.

## Conclusion

When an epidemic breaks out, a vaccine, if available, is one of the most useful and effective tools to control and even eliminate the epidemic. With the development of science and technology, the speed of vaccine research and development is becoming increasingly fast. However, in the early stage of vaccine production, the production capacity is low, and the vaccine supply is limited. Thus, policy-makers must decide how to allocate insufficient vaccines among different age groups or risk groups. Many researchers have investigated this important issue; however, most considered different optimization objectives and modeled vaccine allocation optimization as a single objective optimization problem but ignored the trade-off among different optimization objectives.

Acknowledging the shortcomings of past studies, we proposed a new framework to solve the problem of vaccine allocation optimization. In this framework, our contribution is three-fold. Firstly, we consider allocating vaccines among different age groups and modeled this process as a two-objective optimization problem to consider the two optimization objectives simultaneously and discover the trade-off relationship between them. If necessary, the model can be extended to more than two optimization objectives. Secondly, multistage allocation was also considered to model the scenario where the vaccines are allocated during an epidemic and to observe how the allocation stage impacts the optimal allocation strategy and show the dynamic change of the allocation strategy. Thirdly, to evaluate the outcomes of the different strategies, an age-stratified deterministic extended SEIR model was established. The social contact pattern is contained in the model to show how an infectious disease spreads among different age groups. We applied the framework to the COVID-19 vaccine allocation under the different combinations of vaccine parameters and observed some meaningful results; readers can see more details in the section Results and Discussion. Additionally, the framework can be applied to other vaccine allocation situations by changing the evaluation model's parameters or optimizing the objective as their needs.

There are several limitations of this study. First, we investigated vaccine allocation optimization under a fixed social contact pattern. In reality, when an epidemic breaks out, authorities may make policies to let citizens maintain social contact distance, wear masks, and so on to control the spread of the epidemic. Thus, the social contact pattern is dynamic in an epidemic. In addition to age factor, the other factors, such as occupation (42), sex, chronic diseases (16), may relate to susceptibility rate and infected fatality rate, and further influence vaccine allocation. Integrating these factors in this model requires more professional knowledge about COVID-19. These issues should be considered in future work to obtain more realistic results. Second, how to model the effect of a vaccine should be investigated in more detail. In this study, vaccine can decrease the probability of being infected was assumed and vaccines' effect last longer than the time horizon we study, which was simplistic. However, the immunity waning may exist for some vaccines. Third, due to data unavailability, the case study in this study was simulation-based. Otherwise, the test techniques may be immature in some areas at the early stage of vaccine allocation. If these tests are not performed, it is sure that the asymptomatic individuals have opportunity to get vaccine. We leave this scenario as an important consideration for future research.

## Data availability statement

The original contributions presented in the study are included in the article/Supplementary material, further inquiries can be directed to the corresponding author/s.

## Author contributions

HW and KW contributed to conception and design of the study. HW performed the data analysis and wrote the first draft of the manuscript. All authors contributed to manuscript revision, read, and approved the submitted version.

## Conflict of interest

The authors declare that the research was conducted in the absence of any commercial or financial relationships that could be construed as a potential conflict of interest.

## Publisher's note

All claims expressed in this article are solely those of the authors and do not necessarily represent those of their affiliated organizations, or those of the publisher, the editors and the reviewers. Any product that may be evaluated in this article, or claim that may be made by its manufacturer, is not guaranteed or endorsed by the publisher.

## Supplementary material

Supplementary Figure S1 Pareto optimal fronts when *V*_*e*_ = 20%.

Supplementary Figure S2 Pareto optimal fronts when *V*_*e*_ = 30%.

Supplementary Figure S3 Pareto optimal fronts when *V*_*e*_ = 40%.

Supplementary Figure S4 Pareto optimal fronts when *V*_*e*_ = 50%.

Supplementary Figure S5 Pareto optimal fronts when *V*_*e*_ = 60%.

Supplementary Figure S6 Pareto optimal fronts when *V*_*e*_ = 70%.

Supplementary Figure S7 Pareto optimal fronts when *V*_*e*_ = 80%.

Supplementary Figure S8 Pareto optimal fronts when *V*_*e*_ = 90%.

Supplementary Figure S9 Heatmaps of Pareto optimal solutions when *V*_*e*_ = 20%.

Supplementary Figure S10 Heatmaps of Pareto optimal solutions when *V*_*e*_ = 30%.

Supplementary Figure S11 Heatmaps of Pareto optimal solutions when *V*_*e*_ = 40%.

Supplementary Figure S12 Heatmaps of Pareto optimal solutions when *V*_*e*_ = 50%.

Supplementary Figure S13 Heatmaps of Pareto optimal solutions when *V*_*e*_ = 60%.

Supplementary Figure S14 Heatmaps of Pareto optimal solutions when *V*_*e*_ = 70%.

Supplementary Figure S15 Heatmaps of Pareto optimal solutions when *V*_*e*_ = 80%.

Supplementary Figure S16 Heatmaps of Pareto optimal solutions when *V*_*e*_ = 90%.

Supplementary Figure S17 ICU cases prevention comparation between chosen optimal strategy and baseline.

Supplementary Figure S18 Deaths prevention comparation between chosen optimal strategy and baseline.

Supplementary Figure S19 Pareto optimal fronts of model extension when *V*_*e*_ = 30%.

Supplementary Figure S20 Pareto optimal fronts of model extension when *V*_*e*_ = 60%.

Supplementary Figure S21 Pareto optimal fronts of model extension when *V*_*e*_ = 90%.

Supplementary Figure S22 Heatmaps of Pareto optimal solutions of model extension when *V*_*e*_ = 30%.

Supplementary Figure S23 Heatmaps of Pareto optimal solutions of model extension when *V*_*e*_ = 60%.

Supplementary Figure S24 Heatmaps of Pareto optimal solutions of model extension when *V*_*e*_ = 90%.
