# Supplementary figures and images for: How can age-based vaccine allocation strategies be optimized? A multi-objective optimization framework
Source: Front Public Health. 2022 Sep 8;10:934891. doi: 10.3389/fpubh.2022.934891 (PMC9493087; doi:10.3389/fpubh.2022.934891)

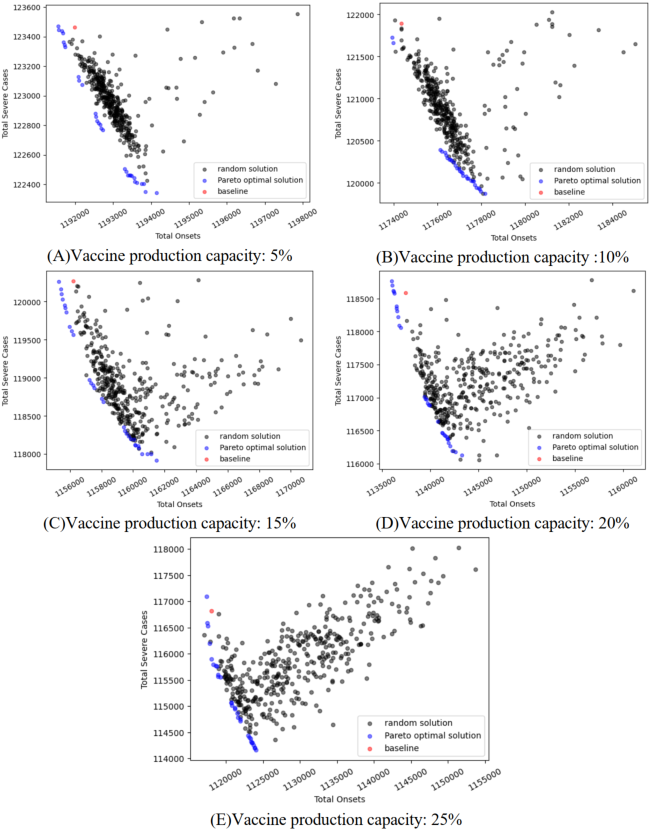

Supplement: Supplementary Figure S1 — Pareto optimal fronts when Ve = 20%. [file Data_Sheet_1.ZIP › supplementary material/Figure S1.jpg]

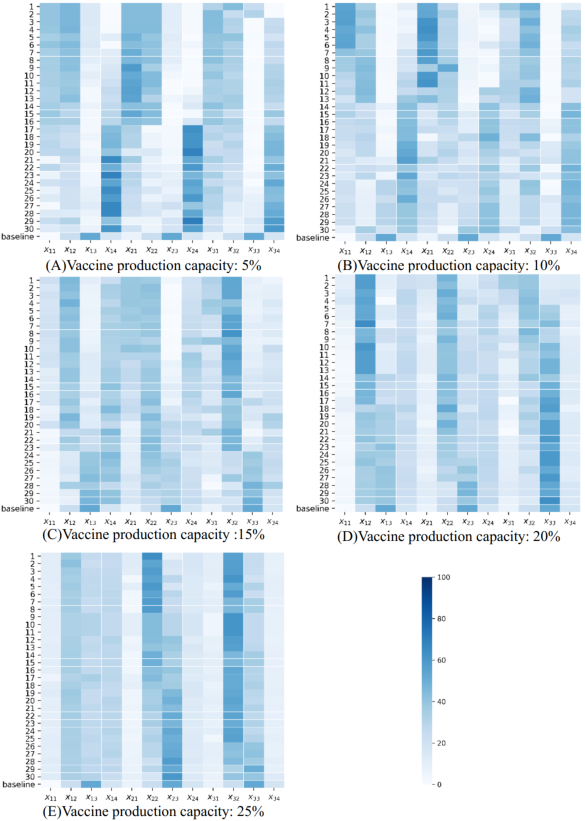

Supplement: Supplementary Figure S1 — Pareto optimal fronts when Ve = 20%. [file Data_Sheet_1.ZIP › supplementary material/Figure S10.jpg]

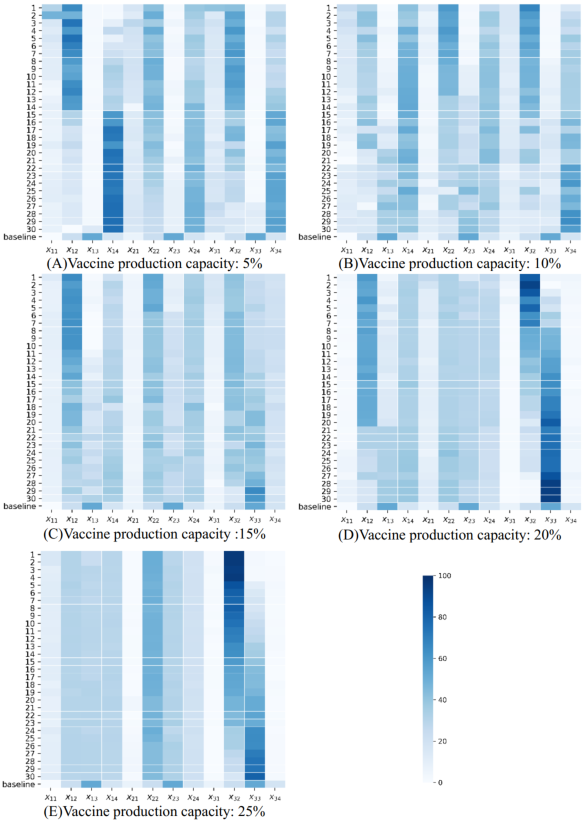

Supplement: Supplementary Figure S1 — Pareto optimal fronts when Ve = 20%. [file Data_Sheet_1.ZIP › supplementary material/Figure S11.jpg]

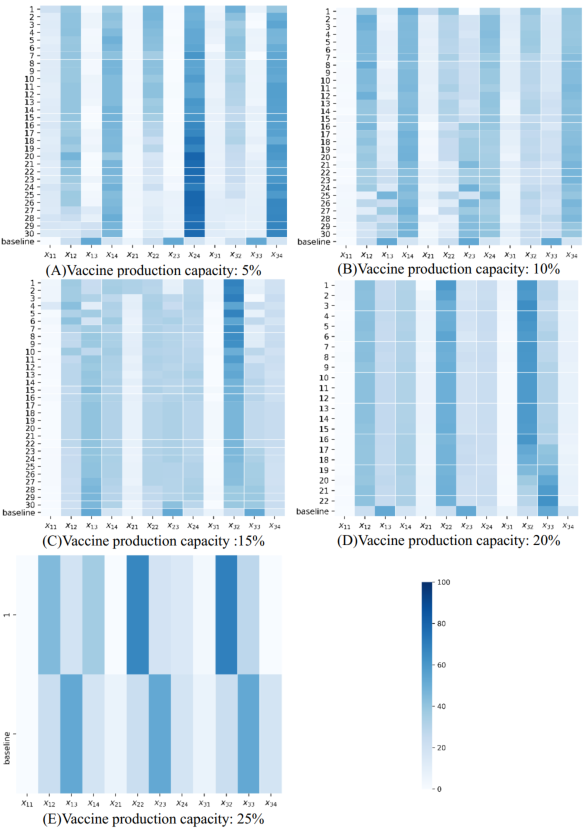

Supplement: Supplementary Figure S1 — Pareto optimal fronts when Ve = 20%. [file Data_Sheet_1.ZIP › supplementary material/Figure S12.jpg]

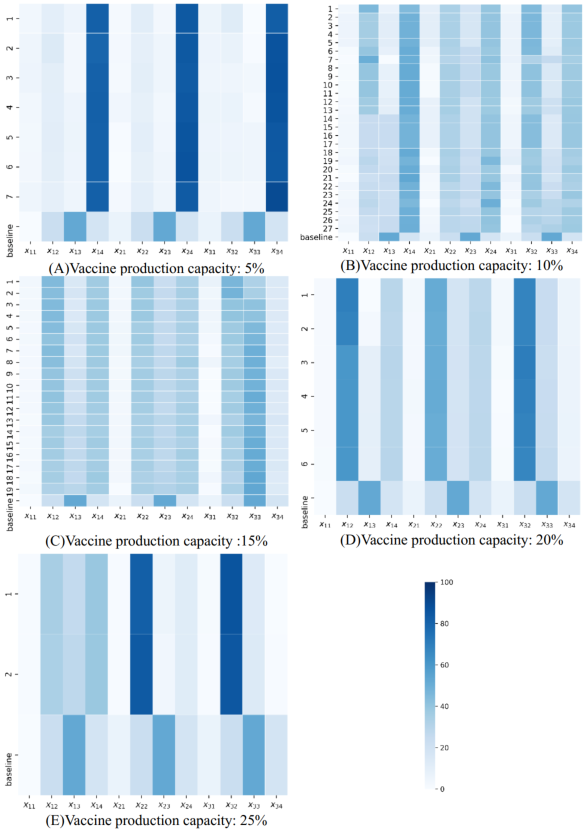

Supplement: Supplementary Figure S1 — Pareto optimal fronts when Ve = 20%. [file Data_Sheet_1.ZIP › supplementary material/Figure S13.jpg]

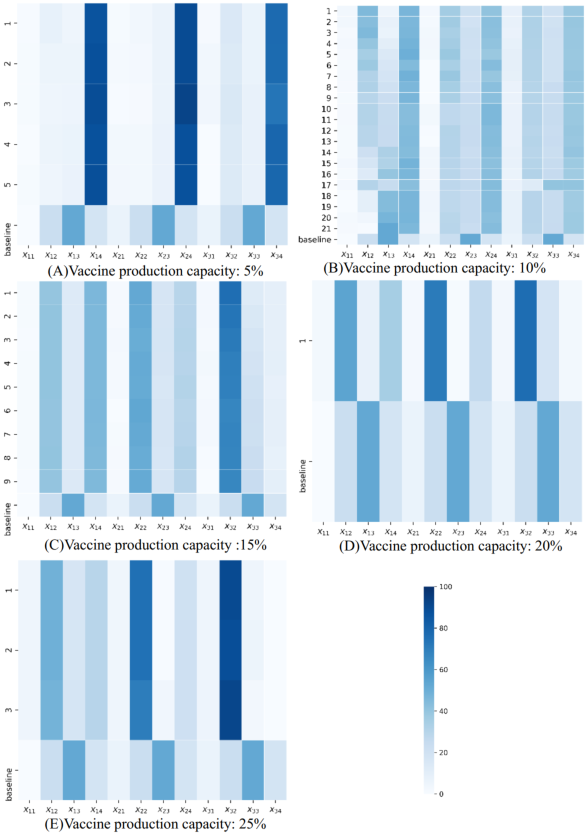

Supplement: Supplementary Figure S1 — Pareto optimal fronts when Ve = 20%. [file Data_Sheet_1.ZIP › supplementary material/Figure S14.jpg]

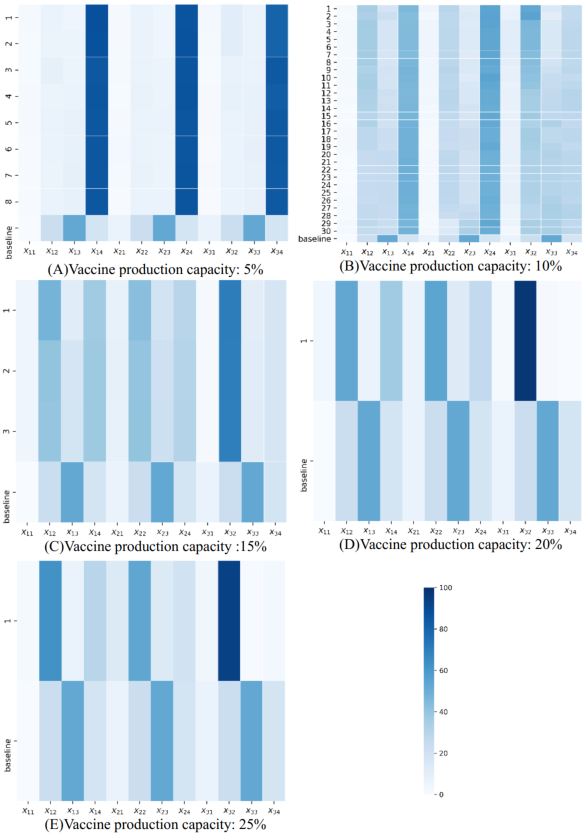

Supplement: Supplementary Figure S1 — Pareto optimal fronts when Ve = 20%. [file Data_Sheet_1.ZIP › supplementary material/Figure S15.jpg]

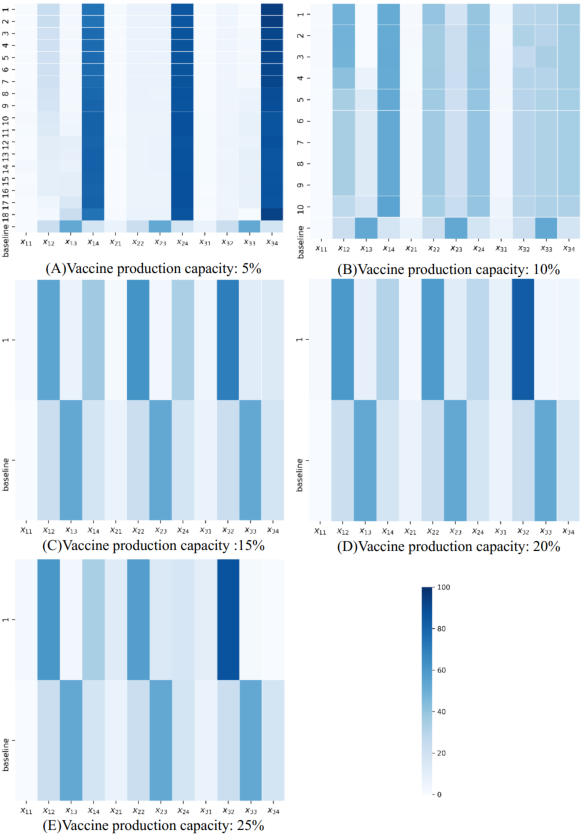

Supplement: Supplementary Figure S1 — Pareto optimal fronts when Ve = 20%. [file Data_Sheet_1.ZIP › supplementary material/Figure S16.jpg]

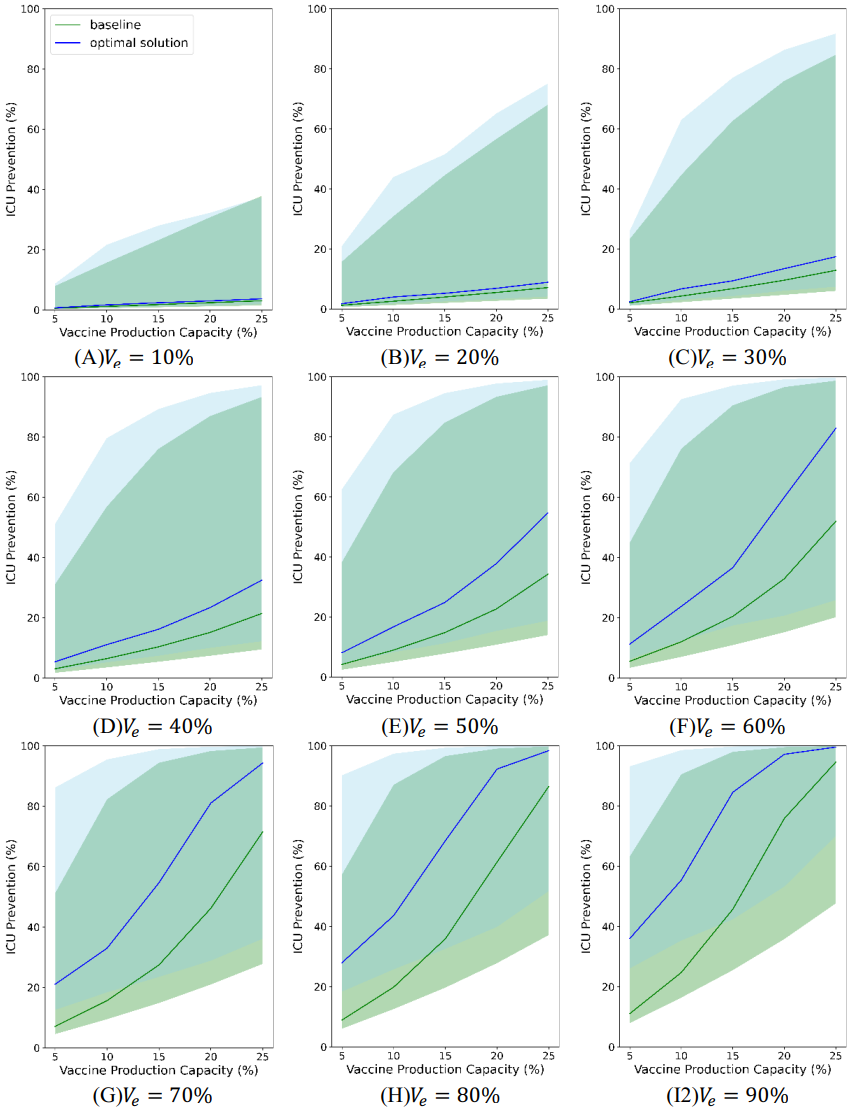

Supplement: Supplementary Figure S1 — Pareto optimal fronts when Ve = 20%. [file Data_Sheet_1.ZIP › supplementary material/Figure S17.jpg]

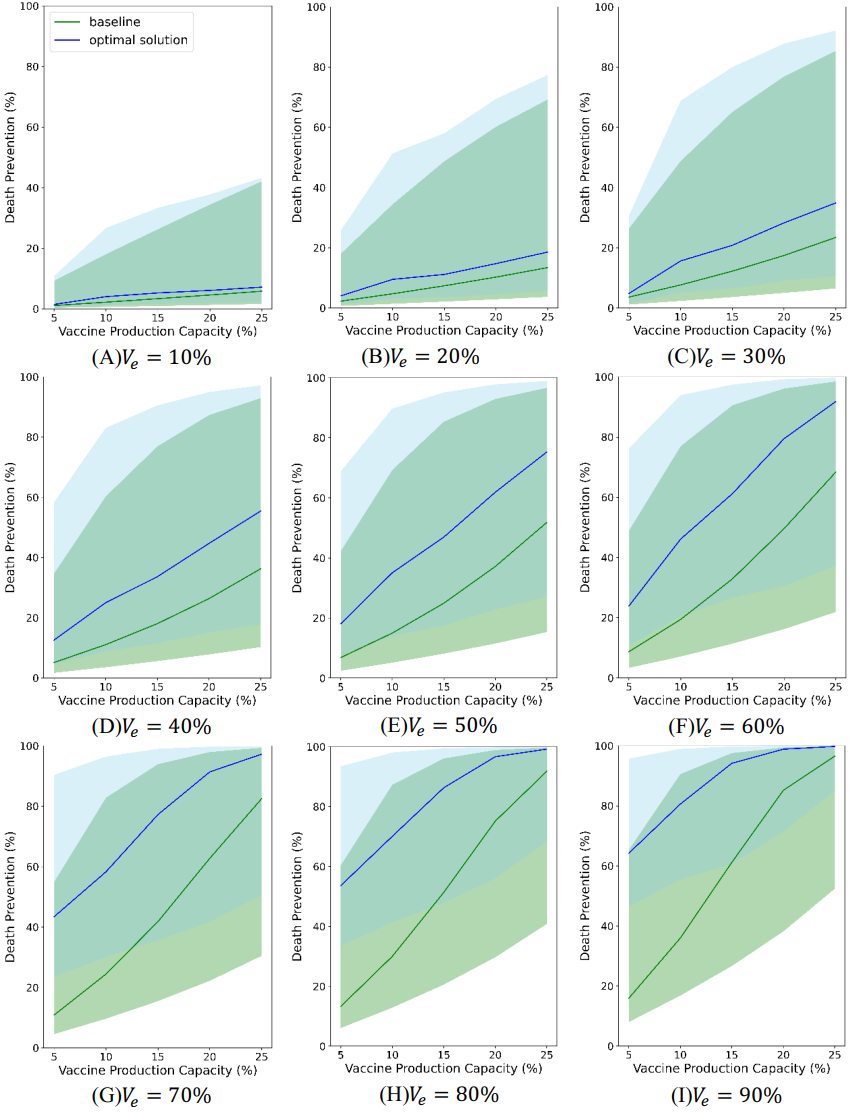

Supplement: Supplementary Figure S1 — Pareto optimal fronts when Ve = 20%. [file Data_Sheet_1.ZIP › supplementary material/Figure S18.jpg]

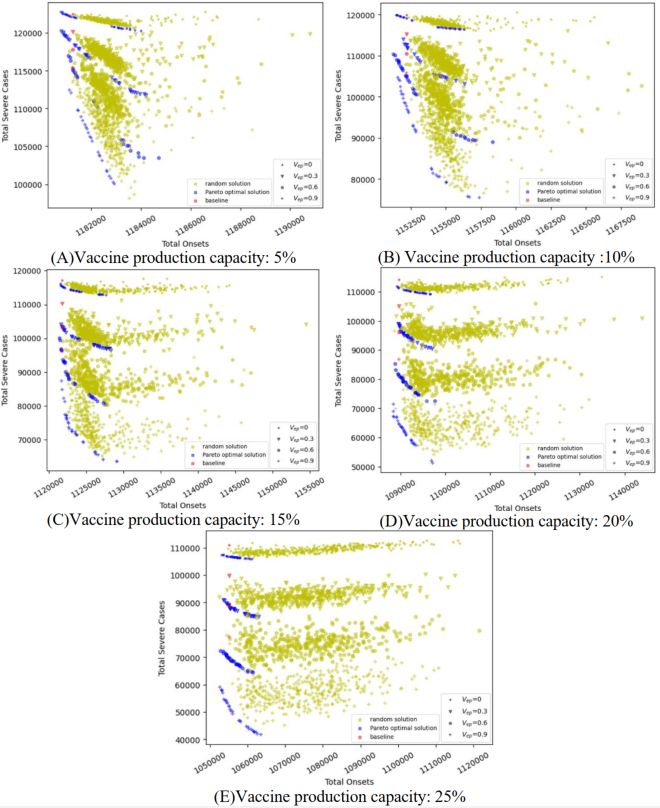

Supplement: Supplementary Figure S1 — Pareto optimal fronts when Ve = 20%. [file Data_Sheet_1.ZIP › supplementary material/Figure S19.jpg]

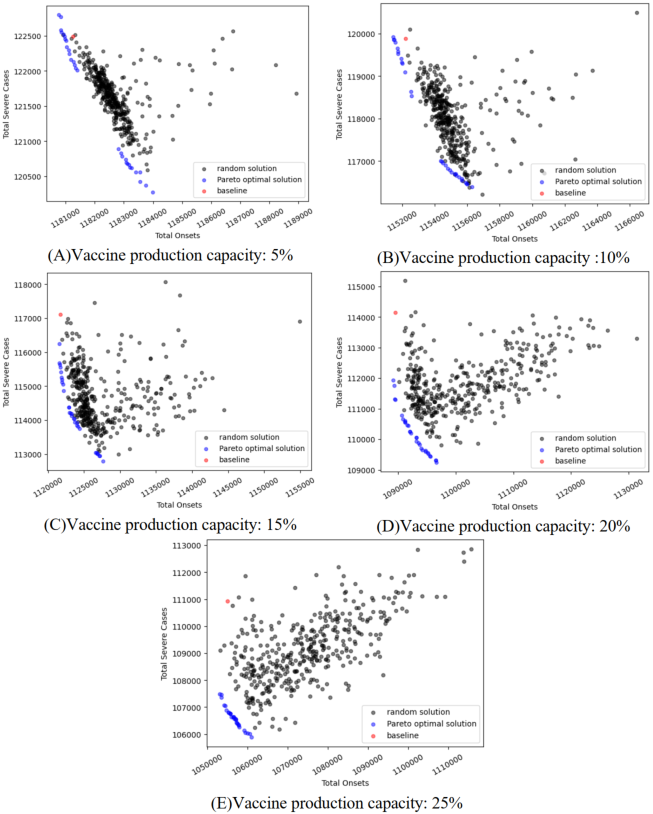

Supplement: Supplementary Figure S1 — Pareto optimal fronts when Ve = 20%. [file Data_Sheet_1.ZIP › supplementary material/Figure S2.jpg]

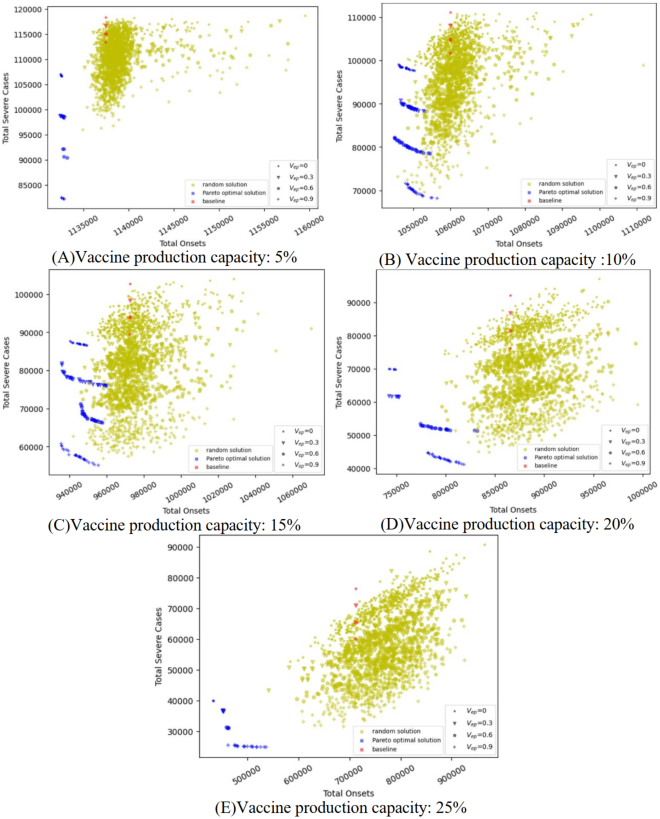

Supplement: Supplementary Figure S1 — Pareto optimal fronts when Ve = 20%. [file Data_Sheet_1.ZIP › supplementary material/Figure S20.jpg]

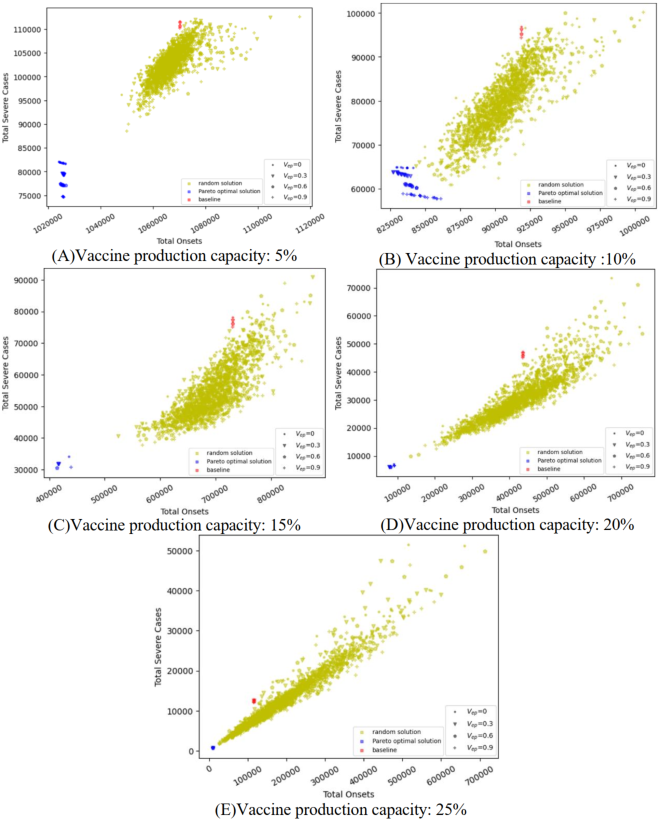

Supplement: Supplementary Figure S1 — Pareto optimal fronts when Ve = 20%. [file Data_Sheet_1.ZIP › supplementary material/Figure S21.jpg]

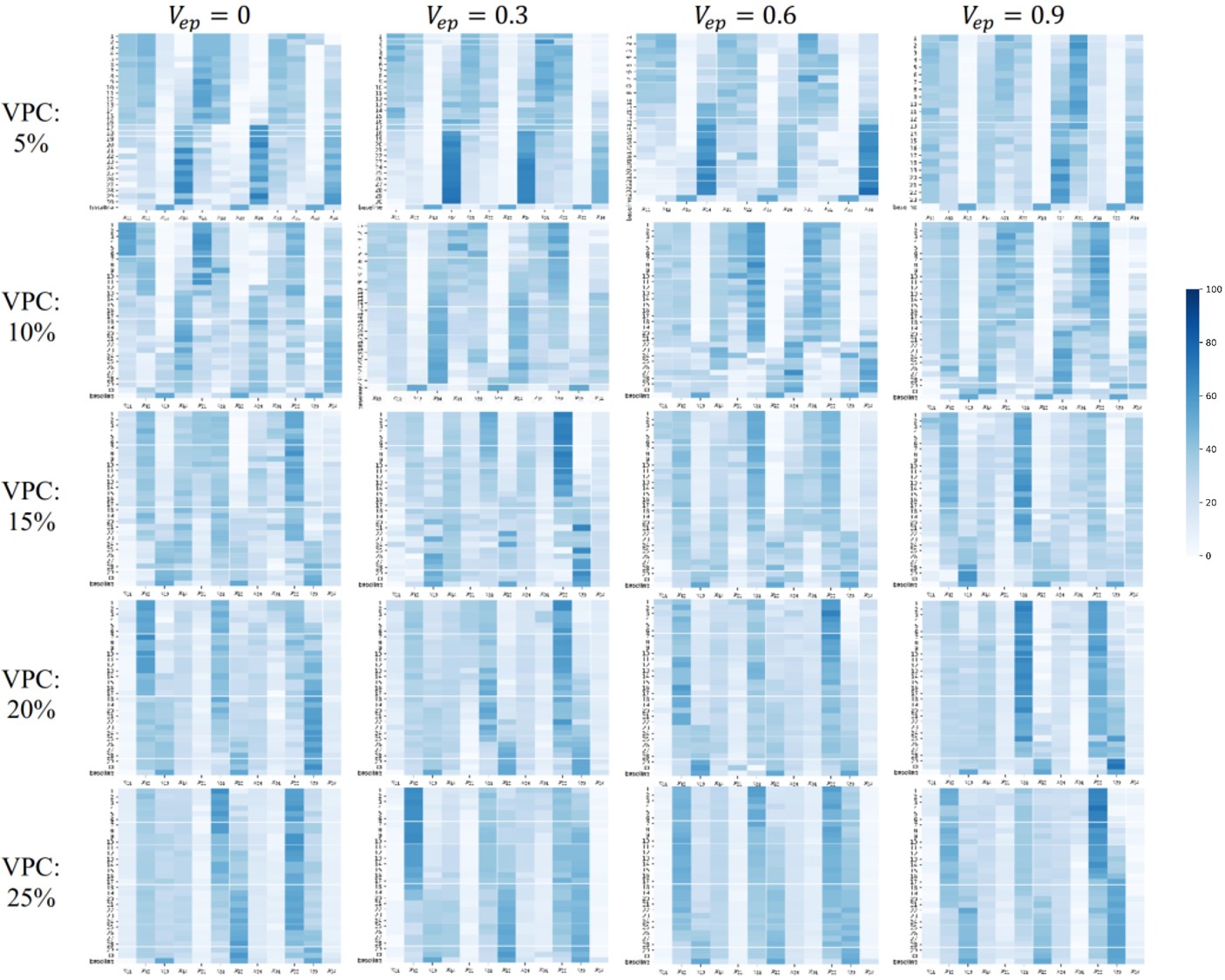

Supplement: Supplementary Figure S1 — Pareto optimal fronts when Ve = 20%. [file Data_Sheet_1.ZIP › supplementary material/Figure S22.jpg]

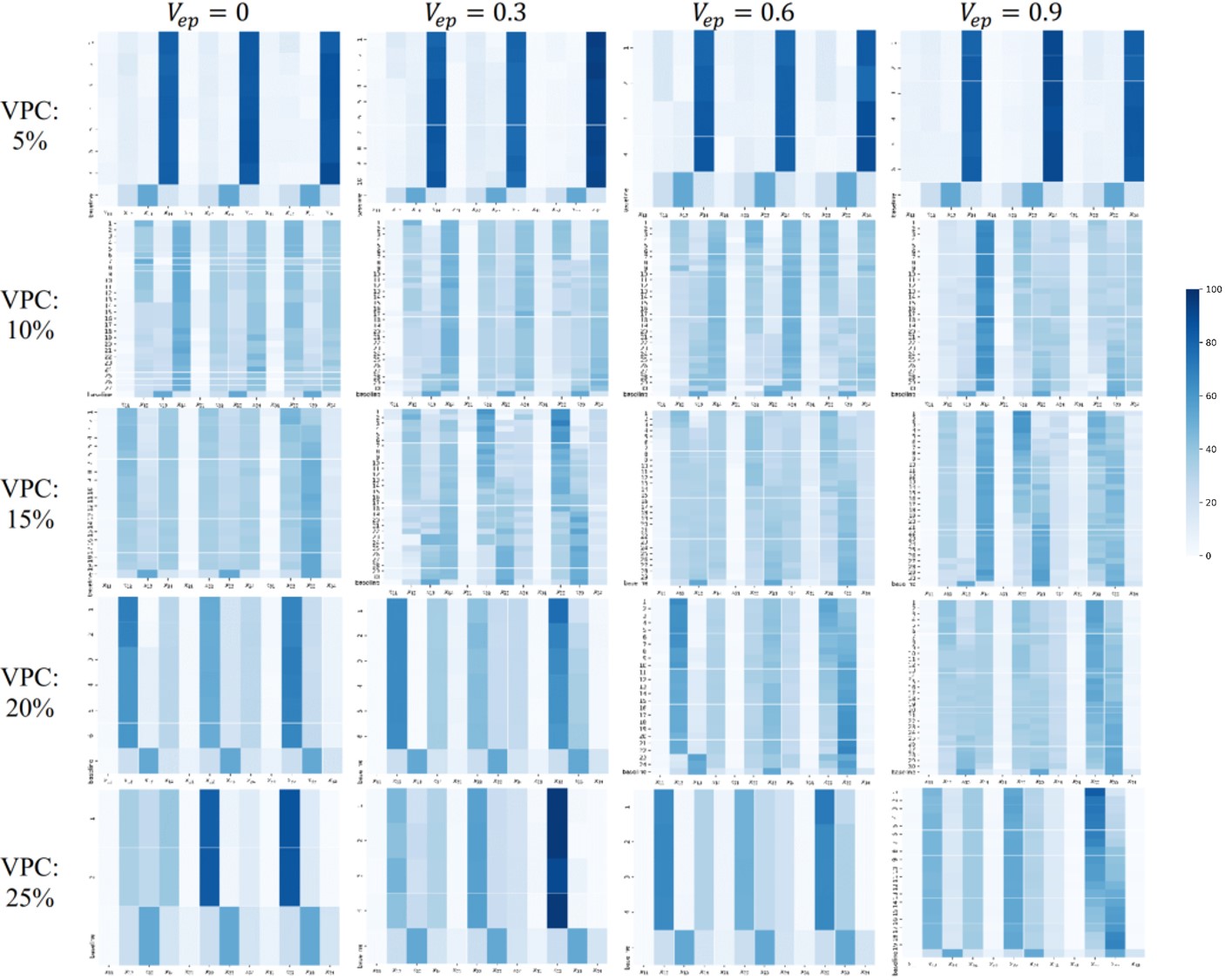

Supplement: Supplementary Figure S1 — Pareto optimal fronts when Ve = 20%. [file Data_Sheet_1.ZIP › supplementary material/Figure S23.jpg]

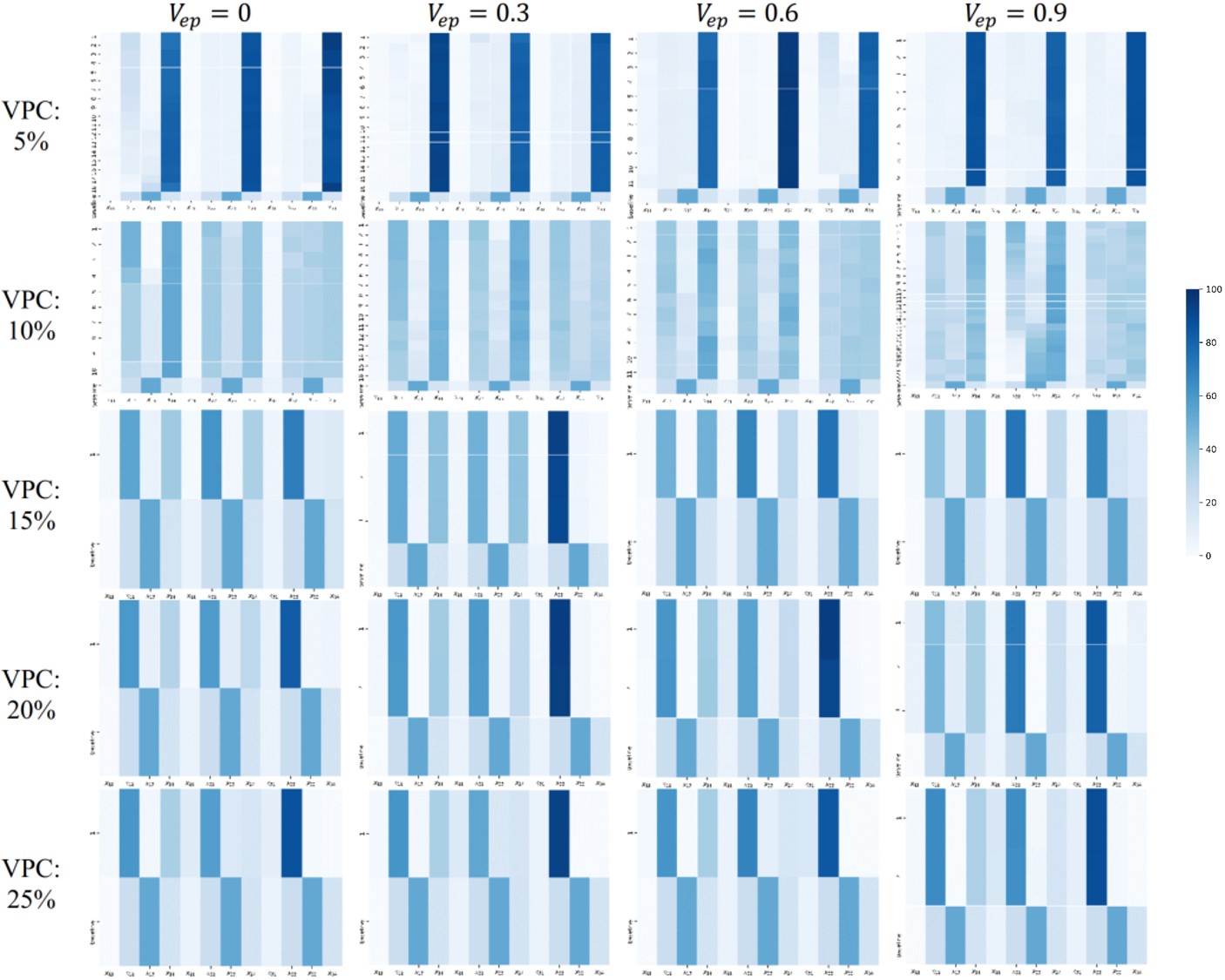

Supplement: Supplementary Figure S1 — Pareto optimal fronts when Ve = 20%. [file Data_Sheet_1.ZIP › supplementary material/Figure S24.jpg]

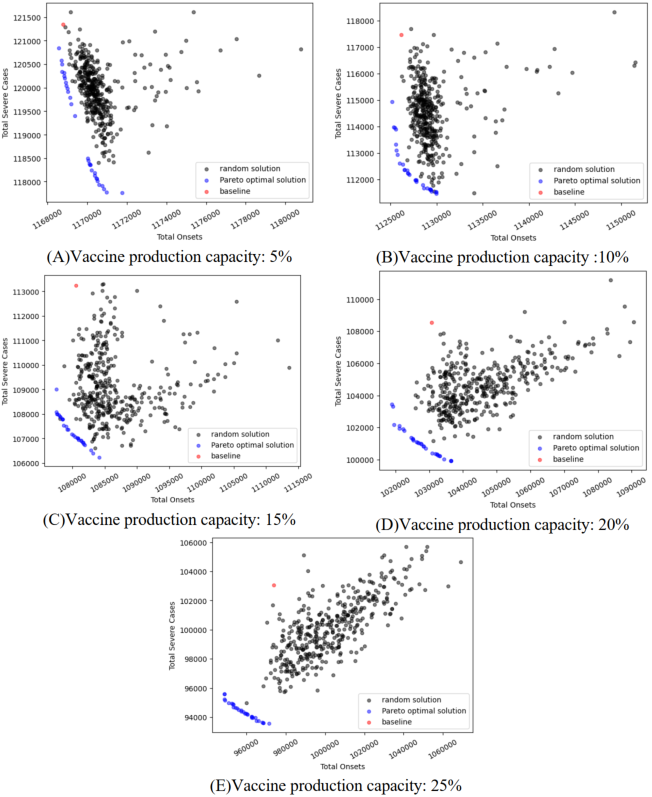

Supplement: Supplementary Figure S1 — Pareto optimal fronts when Ve = 20%. [file Data_Sheet_1.ZIP › supplementary material/Figure S3.jpg]

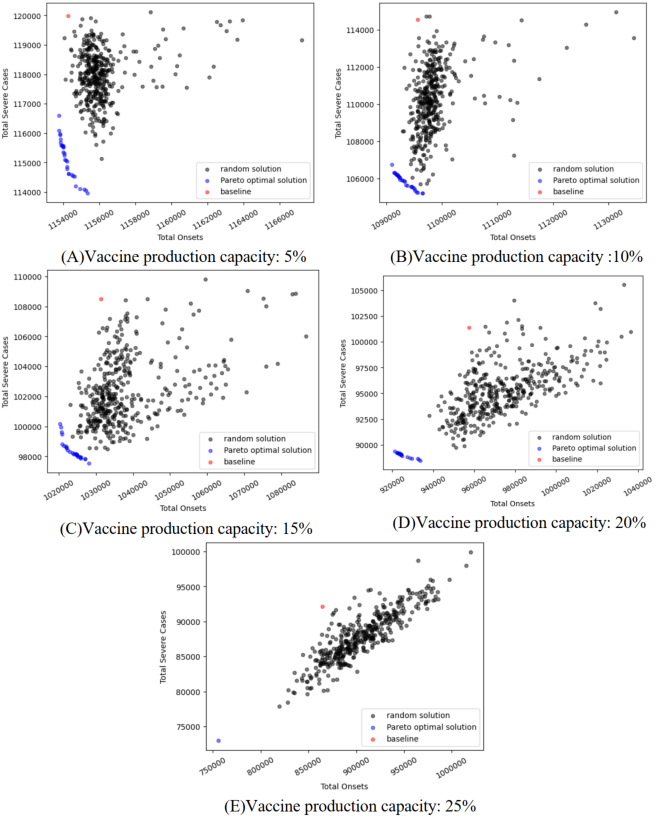

Supplement: Supplementary Figure S1 — Pareto optimal fronts when Ve = 20%. [file Data_Sheet_1.ZIP › supplementary material/Figure S4.jpg]

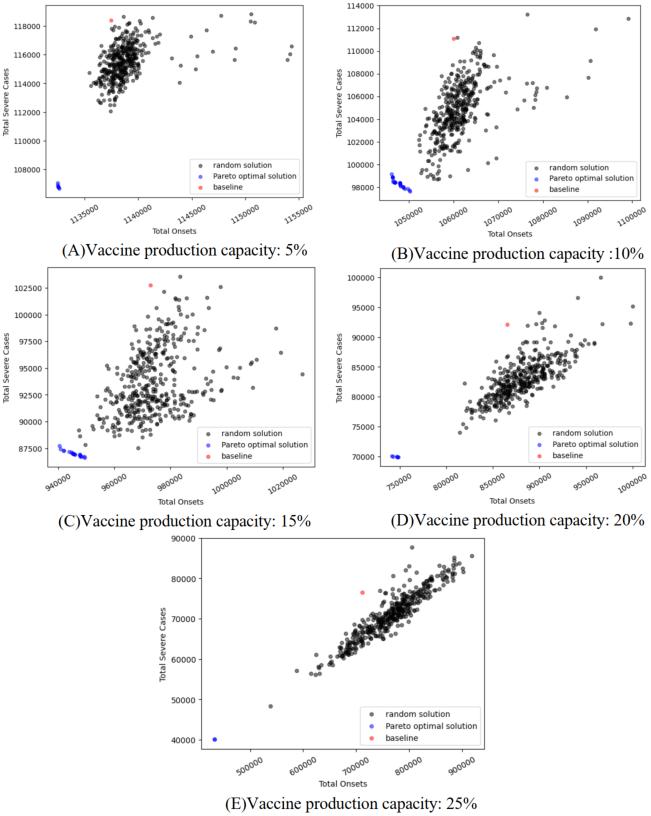

Supplement: Supplementary Figure S1 — Pareto optimal fronts when Ve = 20%. [file Data_Sheet_1.ZIP › supplementary material/Figure S5.jpg]

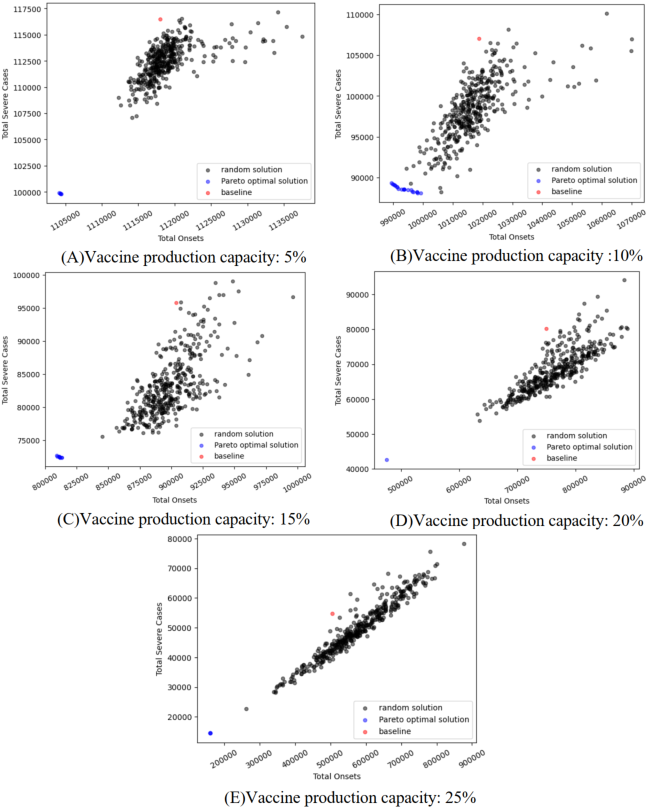

Supplement: Supplementary Figure S1 — Pareto optimal fronts when Ve = 20%. [file Data_Sheet_1.ZIP › supplementary material/Figure S6.jpg]

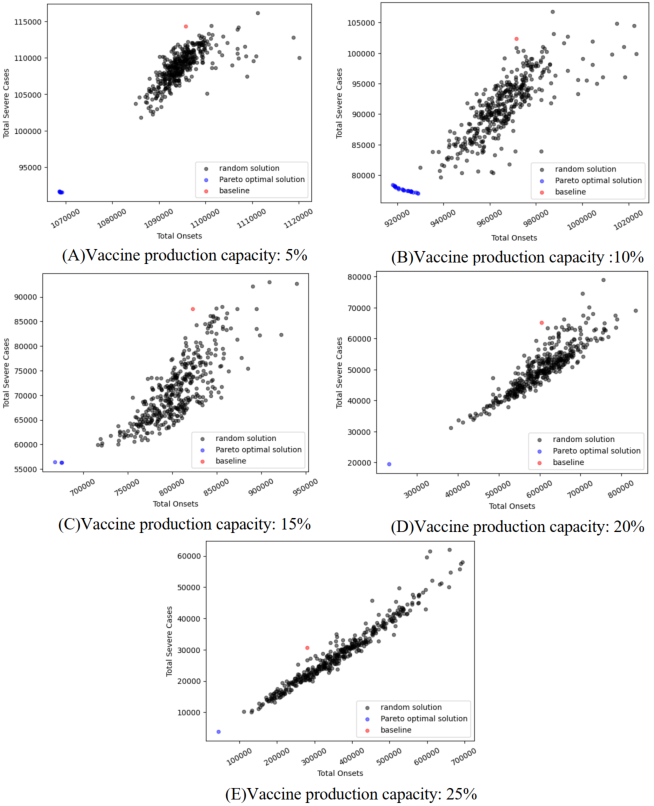

Supplement: Supplementary Figure S1 — Pareto optimal fronts when Ve = 20%. [file Data_Sheet_1.ZIP › supplementary material/Figure S7.jpg]

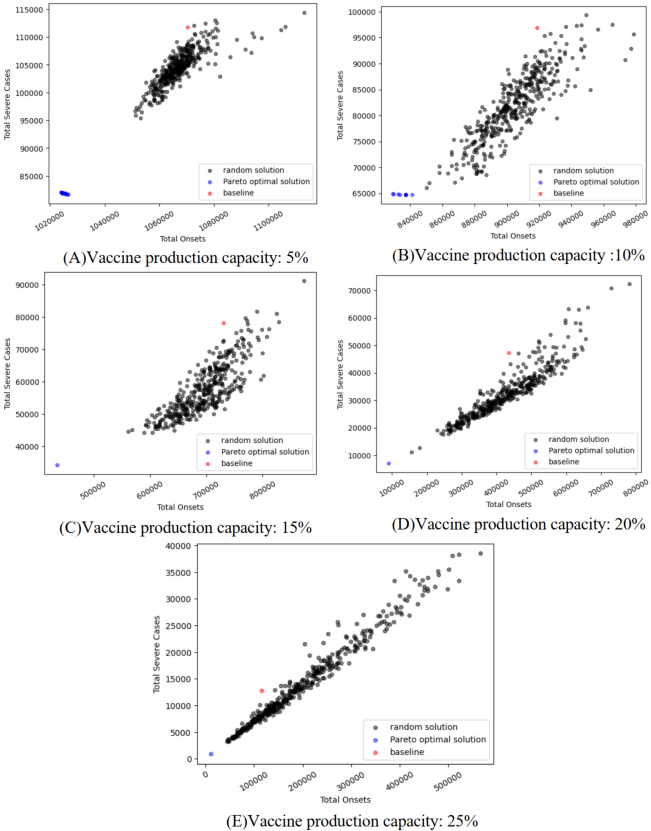

Supplement: Supplementary Figure S1 — Pareto optimal fronts when Ve = 20%. [file Data_Sheet_1.ZIP › supplementary material/Figure S8.jpg]

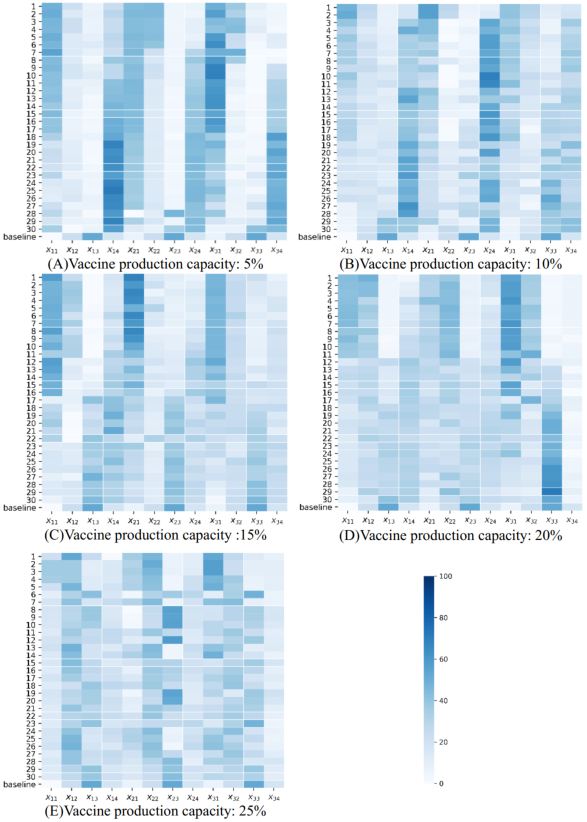

Supplement: Supplementary Figure S1 — Pareto optimal fronts when Ve = 20%. [file Data_Sheet_1.ZIP › supplementary material/Figure S9.jpg]
